# A Novel Approach to Teaching Fundoscopy Using a Virtual Format

**DOI:** 10.15766/mep_2374-8265.11252

**Published:** 2022-05-27

**Authors:** Caroline Vloka, Peter Wingrove, Joshua Ong, Zachary Koretz, Sanya Yadav, Aidan Dmitriev, Levi Bowers, Tyler Miller, Keerthana Samanthapudi, Oliver Beale, Evan Waxman

**Affiliations:** 1 Third-Year Resident, Department of Ophthalmology, University of Pittsburgh Medical Center; 2 Fourth-Year Medical Student, University of Pittsburgh School of Medicine; 3 Second-Year Medical Student, University of Pittsburgh School of Medicine; 4 Residency Program Director and Associate Professor, Department of Ophthalmology, University of Pittsburgh Medical Center

**Keywords:** Fundoscopy, Fundus Photography, Ophthalmoscopy, Ophthalmology, Virtual Learning

## Abstract

**Introduction:**

Ophthalmology education has been underemphasized in medical school curricula despite the fact that patient eye-related complaints are commonplace across primary care specialties. Although previous curricula used direct ophthalmoscopy to teach medical students the fundamentals of ophthalmic examination, there has been a growing call to teach these fundamentals through reading fundus photos due to the increasing prevalence and decreased costs of fundus cameras in primary care settings. We developed a virtual workshop to teach ophthalmoscopy to medical students using fundus photography.

**Methods:**

First-year medical students were enrolled in a 2-hour, synchronous, virtual ophthalmoscopy workshop as part of an advanced physical exam curriculum at the University of Pittsburgh School of Medicine. Students participated in a pretest, introductory lecture, interactive small-group session, and posttest. Breakout groups were led by senior medical students or residents. We compared pre- and posttest results for improved understanding of concepts covered in the workshop.

**Results:**

Of 147 students, the average scores on the pretest and posttest were 39% and 75%, respectively (*p* < .01). Students were significantly more confident in their ability to identify various pathologies on fundus photography. After the workshop, the student preceptors indicated increased comfort in a teaching role and greater interest in medical education. The preceptors were also more confident in their own ability to interpret fundus photography and in their understanding of various ocular pathologies.

**Discussion:**

Our virtual, interactive workshop is effective in teaching medical students a systematic approach to the interpretation of fundus photographs.

## Educational Objectives

By the end of this activity, learners will be able to:

1. Use a systematic approach to evaluate fundus photographs.
2. Identify all components of a fundus photograph and describe the characteristics of the normal ocular fundus.
3. Identify normal variants of the ocular fundus.
4. Identify various pathologies commonly found on a fundoscopic exam and make connections to associated diseases (e.g., diabetes, hypertension, elevated intracranial pressure, glaucoma).

## Introduction

Ophthalmology education has been underemphasized in medical school curricula.^1–4^ One study noted that most primary care program directors believe a majority of their incoming residents do not meet the standard knowledge expectations laid out by the Association of University Professors of Ophthalmology.^3^ This is despite the fact that patient eye-related complaints are commonplace across many primary care specialties in both the inpatient and outpatient settings.^4,5^

The International Council of Ophthalmology has proposed a competency-based approach to ophthalmologic education to prepare medical students for the management of patients with eye complaints. Its recommendations include teaching the clinical skills needed to assess visual acuity, visual fields, extraocular movement, and ancillary signs of eye health.^6^ Additionally, emphasis has been placed on knowledge of ocular anatomy as well as medical understanding of ophthalmic diseases and ocular manifestations of systemic diseases.

Previous curricula used direct ophthalmoscopy to teach medical students the fundamentals of ophthalmic examination. However, given the infrequency of its use in practice, alongside its low accuracy in detecting pathology,^7^ the utility of this teaching method has been called into question.^8,9^ Instead, there has been a growing call to teach a systematic approach to how to read a fundus photo and assess for pathology given the increasing prevalence and decreased costs of fundus cameras in primary care settings.^4^ Biousse and colleagues highlighted this point, arguing that visualizing the fundus is more important than the method used, thus allowing for more nonophthalmologists to take part in screening for eye disease.^9^

To meet the recommendations set out by the International Council of Ophthalmology, medical students at the University of Pittsburgh School of Medicine (UPSOM) are provided with a comprehensive clinical ophthalmology curriculum.^10^ The first part of the students’ ophthalmological education is in the form of a 3-hour ophthalmoscopy skills workshop conducted during the first year of medical school. This workshop is part of the required advanced physical examination (APE) course, which is designed to help learners develop history taking and physical exam skills. These sessions are typically conducted in person; however, in light of the COVID-19 pandemic, they were transitioned to a virtual platform. We developed a 2-hour, synchronous, virtual ophthalmoscopy workshop as part of the APE curriculum for first-year medical students at UPSOM to substitute for the traditional ophthalmoscopy skills session.

Our workshop is a stand-alone, image-rich, interactive module for interpreting fundus photographs. To our knowledge, there are currently no publications in *MedEdPORTAL* or elsewhere describing the use of a virtual platform for the purpose of teaching fundoscopic eye exams to medical students.

## Methods

The workshop consisted of a 10-minute pretest, a 30-minute introductory lecture, a 70-minute interactive interpretation session, and a 10-minute posttest. Senior medical students and ophthalmology residents prepared all lecture slides and evaluations with the oversight of an ophthalmology attending.

Prior to the workshop, the administrators divided the first-year medical student class into three groups of 50 students, each of which participated in one of three sessions administered over 3 consecutive days.

At the beginning of each session, we asked the participating students to open a link shared in a Zoom chat that led to a pretest administered via Google Forms (Appendix A). Students were given 10 minutes to complete the test. They were then asked to activate their video and tune in to the introductory lecture, which was presented by either an attending or senior resident (Appendix B, slides 1–21).

The introductory lecture delineated the workshop's learning objectives and introduced the basic anatomy and physiology of the optic nerve, retinal vessels, macula, and retina. Additionally, we described a systematic approach to the interpretation of fundus photography. Toward the end of the lecture, the presenter went through two examples (one normal and one pathologic image) of how to systematically interpret fundus photos (Table 1).

**Table 1.**
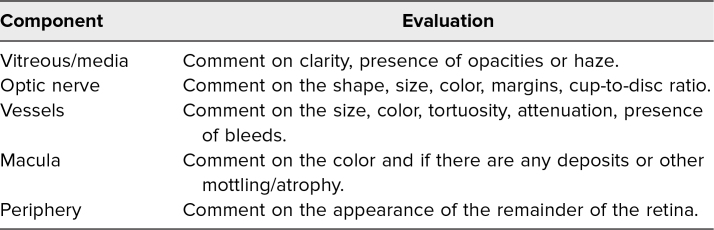
Systematic Approach to Evaluating the Components of the Fundus

For the interactive interpretation sessions, which were led by preceptors, we divided students into groups of four to five using the breakout room function on Zoom. The preceptors aimed to complete at least 15–20 slides within the breakout groups. There were extra slides provided if any group had extra time.

Within the breakout groups, the preceptor picked an order in which the medical students were expected to participate. We asked each student to evaluate one fundus photograph in the systematic way that had been described in the introductory lecture. Students took turns interpreting these slides in the predetermined order. On each slide, there was a photo on the right for the student to interpret alongside a photo on the left that acted as a normal control for comparison. At least one attending was present during each of the three sessions and rotated through the breakout rooms to oversee progress. Though attendings acted mostly in a supervisory role, they provided insight and clarification when necessary.

In order to reinforce an organized and systematic approach to the photo interpretation, we asked students to verbalize their analysis of the media, nerve, vessels, macula, and retina for each photo. To introduce pathologies, the preceptor provided information on clinical correlates associated with the findings. In the latter half of the breakout session, we asked the students to comment on whether they thought the various components of the fundus exam were normal and to reflect on potential etiologies for the pathologic findings.

After the allotted 70 minutes, we closed the breakout groups, and all participants returned to the main Zoom session. Another link was then posted into the chat that led students to the 10-minute posttest (Appendix C). After completing the posttest, students were free to leave the session.

### Learner Evaluation

The pre- and posttests were anonymous and only used as an assessment of the baseline knowledge of the group of students and as a measure of the effectiveness of the workshop. The assessments were not used to determine a grade for any individual student. Instead of names, participants were asked to create a unique identifier consisting of their favorite color and the last three digits of their cell phone number. We asked students to interpret the relevant findings and to answer related questions.

The pre- and posttests were developed by the same medical students and residents who were involved in preparing the slides for the workshop. The group worked collectively to identify the normal fundus and pathologies that were most useful in the assessment of the fundus. The 15 test questions, consisting of fundus photographs, were in an identical order to allow valid comparison between the pre- and posttest results.

In both the pre- and posttests, we asked students to rate their confidence in their ability to interpret various components of fundus photos using a 7-point Likert scale (1 = *no confidence,* 7 = *extremely confident*). Students were also asked to rate their interest level in ophthalmology as a career using a similar 7-point Likert scale (1 = *minimal,* 7 = *very interested*). Moreover, we collected open-ended responses concerning the most useful aspect of the session and suggestions for improvement. In response to student feedback, we prepared a handout to distribute to students after the completion of the workshop (Appendix D).

We also asked participants if they had any prior ophthalmological experience (in the pretest) and if their small group was led by a student or resident (in the posttest). We analyzed the pre- and posttest data using Stata 15.1 (StataCorp). We made pre- and posttest comparisons, as well as pre- and postworkshop confidence comparisons, using unpaired *t* tests.

### Preceptor Training and Evaluation

The preceptors were either residents or senior medical students (third-year or fourth-year medical students), and at least one attending rotated through the breakout rooms during each of the sessions. If there were more preceptors than breakout rooms, medical student preceptors were paired with residents to comanage the breakout rooms. Medical students were volunteers who were interested in both ophthalmology and medical education. All volunteers were invited to participate in student teaching. We expected the preceptors to understand ocular anatomy, the components of the ophthalmologic examination, and fundus photo interpretation, as well as clinical correlates and pathophysiology of relevant findings on fundoscopy. To further clarify the expectations of student teachers, we prepared a handout with the proficiencies necessary for medical students to act as peer educators (Appendix D).

In order to prepare the preceptors for the breakout rooms, we held two 3-hour review sessions in the week leading up to the workshop to ensure familiarity with the material and a standardized approach to teaching the components of the fundus exam and interpretation. During these sessions, the breakout-room slides (Appendix B, slides 22–43) were analyzed as a group, and the pathophysiology and differential diagnosis for each finding were described. Between group training sessions, medical students and resident preceptors were encouraged to ask questions in our Slack (Slack Technologies) channel to help reinforce their understanding of the material covered. Preceptors were expected to demonstrate understanding of the material through a teach-back method, in which one student would act as a preceptor and another would take the role of the junior medical student under supervision of the senior resident or attending. The training of all medical student preceptors was overseen by a senior ophthalmology attending.

After the workshop, senior medical student preceptors were asked to fill out a survey using a 7-point Likert scale (1 = *disagree completely,* 7 = *completely agree*), evaluating their comfort in teaching and interest in medical education and ophthalmology (Appendix E).

## Results

All first-year medical students at UPSOM (*N* = 147) participated in our workshop in December 2020. Students who did not fill out both the pre- and posttests were excluded from our analysis (*n* = 2). One student completed the posttest 40 minutes after the end of the session and was also excluded from analysis. Data collected from the remaining 144 students were included in the final analysis.

The average scores on the pretest and posttest were 39% and 75%, respectively (*p* < .01). Furthermore, the students’ confidence in their ability to identify various pathologies on fundus photography significantly improved, as shown by an increase in their average ratings (based on a Likert scale) from 8.1 to 24.7 points out of a 35-point maximum (Table 2).

**Table 2.**
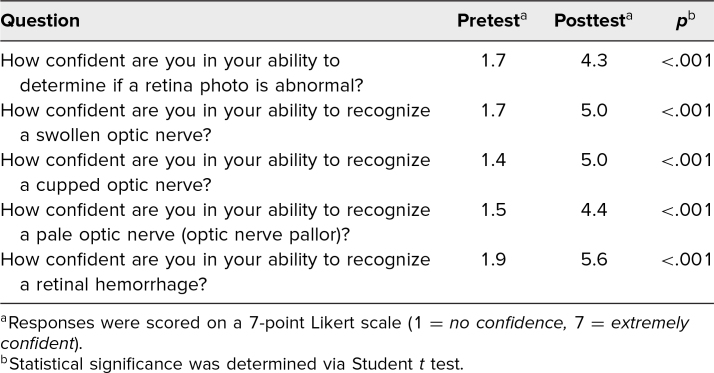
Learners’ Confidence in Identifying Anatomy and Pathology on Fundus Photography Before and After the Workshop (*N* = 144)

Of the 144 students included in the final analysis, 133 responded to the open-ended question “What was the most useful aspect of this APE course?” Of these 133 students, 91% (*n* = 121) responded that the opportunity to go over multiple slides in a small group with a medical student or resident was the best part of the course. Below are some examples of the open-ended responses students submitted regarding feedback about the course:

• “The interactive session in breakout rooms was a good way to stay engaged and was a good change from our usual didactic lectures. I also liked having students explain each image as I think it helped students become more comfortable with the terminology and challenged students to make guesses.”
• “I liked going through so many photos in a very systematic way because I really started to get the hang of identifying normal, abnormal, and normal variations as we went on! Also, I liked how we were in very small groups because it gave us more opportunities to try.”
• “The small groups because it was very interactive.”

Based on open-ended feedback we received on the posttests, participants also enjoyed being taught by their peers:

• “Going through the examples in small groups and working with the MS3 med students. I wasn't afraid to participate and be wrong since they're also students.”
• “Having the senior student walk us through the images.”
• “Just going over a lot of examples and talking out loud with a really open and friendly instructor. Our third-year medical student was great at explaining and conversing with us.”

We did not receive any feedback requesting that students be removed from the role of breakout room preceptors.

Students were also asked, “What suggestions do you have for next year's APE session?” One hundred fourteen participants responded. A majority of the suggestions centered around timing and the pace of the session. However, there was no consensus, as approximately half the responses called for slower-paced sessions with more allotted time, while the other half recommended a faster pace with shorter sessions. Twenty-five students responded that there was nothing they would change. Some suggested that the virtual format should become standard, even if social distancing is not necessary in the future:

• “No improvements needed. This was one of my favorite courses in med school so far!”
• “I think this was a very well run session, I would continue to have a similar format for future sessions. It was very informative and helpful.”
• “LOVED this course!! learned so much and now love ophtho!”

Thirteen students (10%) responded that an in-person session would improve the workshop by enabling hands-on experience with taking photographs and using ophthalmoscopes. Twelve students (9%) mentioned that they would have appreciated some prereading, a handout, or annotated slides to make the session easier to follow.

Student preceptors included students from the second year (*n* = 1), third year (*n* = 6), and fourth year (*n* = 3) of medical school. Volunteers taught an average of 2.67 sessions. After the sessions, the student preceptors reported that they were more comfortable in a teaching role and had a greater interest in both teaching and medical education. For those volunteers who taught more than one session, each felt more confident answering student questions by the third session. Furthermore, the students were also more confident in their own ability to interpret fundus photography and in their understanding of various ocular pathologies.

## Discussion

This workshop was designed to introduce the examination of fundus photos to first-year medical students as a part of the specialty care component of the APE course at UPSOM. The session was held in December 2020 and was the first session of the APE curriculum. The workshop was the first exposure our students had to the interpretation of medical imaging. The workshop emphasized the importance of an organized and systematic approach to the interpretation of any medical imaging or test and applied the strategy to fundus photography.

Our workshop improved medical students’ ability to identify normal anatomy as well as various common pathologies on fundus photos, as exemplified by the dramatic improvement between their pre- and posttest scores. Furthermore, the learners’ confidence in their ability to identify various pathologies also significantly improved. The small-group breakout sessions of four to five students with senior student and resident preceptors allowed for interactive discussion and participation in a low-stress setting. Though the preparation of senior medical students for the role of small-group facilitators required quite a significant amount of time, the endeavor was very well received by the trainees (first-year medical students) and the student teachers (senior medical students). We hope that senior medical students will continue to hold an active and prominent role in running this curriculum in the coming years.

In light of the COVID-19 pandemic and social distancing requirements, the workshop was designed to be conducted virtually and replaced the direct ophthalmoscopy workshop previously taught at UPSOM. Though designed out of necessity, this virtual version of the workshop possesses several advantages over the in-person format. First, in prior years, the focus was primarily on teaching the technique of using a direct ophthalmoscope, which is no longer being utilized in most primary care settings owing to the difficulty associated with its usage.^11,12^ Fundus cameras provide a cheap and feasible alternative and are now gaining popularity among primary care physicians.^13–15^ By prioritizing identification of pathology on fundus photos, the virtual version of the workshop is better suited to equip students with a readily translatable skill that they are more likely to use in their future practice. Additionally, this method serves to introduce the concept of fundoscopy in a tiered approach. By eliminating the challenges presented by the exam itself, this method leaves more time to expose students to a much broader array of pathological pictures. Once the students are trained and feel more confident in recognizing the patterns on preacquired fundus photos, they are more likely to identify pathology through a quick glance afforded by a direct ophthalmoscope, if needed.^12^

Feedback obtained from participants was overwhelmingly positive and indicated that the workshop was engaging and useful for attendees and student teachers alike. In response to student feedback, a handout was prepared and distributed after the completion of the workshop (Appendix D). Additionally, in future years, organizers plan to ask participants to read the first two chapters of *OphthoBook,* by Dr. Timothy Root.^16^ Although a small number of students requested the annotated slides be handed out prior to the workshop, the workshop organizers do not think that would be helpful, as it might deter active participation and independent interpretation of images.

Student teachers also found the sessions helpful, reporting increased interest in medical education, greater confidence in answering student questions, and a better understanding of common ocular pathologies and findings. Peer teaching has been shown to have a generally positive impact on student engagement and understanding of a given topic.^17,18^ Though we did not create or use a formal teaching curriculum for the student leaders, the positive subjective feedback received on the postworkshop survey is similar to results obtained in prior studies.^19^

The organizers found that the most important factor in selection and preparation of student teachers was the interest and motivation of the volunteers. We found that reviewing teaching techniques and methods for keeping students engaged was also useful to the volunteers, as this was the first time many of them were in a teaching position. There was repetition in the questions the student teachers asked, which could have been because not all of them were able to make each review session or because of misunderstandings during the initial presentation. For future courses, we plan on recording review sessions and making them available to the student teachers for review on their own time.

A few months after the session, the organizers of the ophthalmology APE session were invited to present the session again for the Medical Student Second Look Day. This is a day organized by current medical students for recently admitted students to see the opportunities available at UPSOM. The first-year class determined that this teaching session was so engaging and effective that they nominated it as a selling point for potential matriculating medical students.

### Limitations

Our project has several limitations. The tests were designed by the workshop instructors, so it is possible there was some bias of teaching to the test in some sessions. Furthermore, we did not test for delayed recall at any time after the conclusion of the 3-day workshop. Though we sent out a postworkshop test approximately 6 weeks later, there were not enough responses to allow for valid conclusions to be drawn. Finally, we recognize that this workshop focuses primarily on visual identification of fundoscopic features and pathology without a broader patient history, which disengages the identification skills from a clinical context.

### Future Directions

Future opportunities to strengthen this workshop include widening the target audience to include residents in family medicine, internal medicine, or emergency medicine, as well as other practitioners who routinely care for patients with eye or vision complaints. Additionally, though somewhat outside the scope of the current workshop, the same slides could be used to teach the pathophysiology of disease as manifested in fundoscopy. Furthermore, this platform could allow for the expansion of ophthalmology education to medical students at schools that do not have an ophthalmology program or a set ophthalmology curriculum. Finally, we may consider following up the workshop with a weekly, single-image quiz asking participants to interpret an image each week for 4–6 weeks after the completion of the workshop. This would both provide feedback on how well the information is retained and allow the students to continue to implement the learned skills. The scalability of this workshop design is enhanced by the successful and feasible inclusion of student teachers.

### Conclusions

Our virtual, interactive workshop is useful and effective in teaching medical students a systematic approach to the interpretation of fundus photographs. The workshop allows senior medical students to effectively participate as small-group leaders. Statistically significant changes in knowledge and confidence among learners indicate that the workshop is successful in teaching medical students how to identify relevant pathologic findings on fundus imaging.

## Appendices


Pretest.docxSlide Deck.pptxPosttest.docxPostworkshop Handout.pdfMedical Student Session Leader Survey.docx
*All appendices are peer reviewed as integral parts of the Original Publication.*

## References

[1] Mottow-Lippa L. Ophthalmology in the medical school curriculum: reestablishing our value and effecting change. Ophthalmology. 2009;116(7):1235–1236.E1. 10.1016/j.ophtha.2009.01.012 19576494

[2] Quillen DA, Harper RA, Haik BG. Medical student education in ophthalmology: crisis and opportunity. Ophthalmology. 2005;112(11):1867–1868. 10.1016/j.ophtha.2005.05.005 16271315

[3] Stern GA. Teaching ophthalmology to primary care physicians. Arch Ophthalmol. 1995;113(6):722–724. 10.1001/archopht.1995.01100060048029 7786211

[4] Graubart EB, Waxman EL, Forster SH, et al. Ophthalmology objectives for medical students: revisiting what every graduating medical student should know. Ophthalmology. 2018;125(12):1842–1843. 10.1016/j.ophtha.2018.08.032 30454712

[5] Gelston CD, Patnaik JL. Ophthalmology training and competency levels in caring for patients with ophthalmic complaints in United States internal medicine, emergency medicine, and family medicine residents. J Educ Eval Health Prof. 2019;16:25. 10.3352/jeehp.2019.16.25 31461804 PMC6748877

[6] Tso MO, Goldberg MF, Lee AG, Selvarajah S, Parrish RK, Zagorski Z. An international strategic plan to preserve and restore vision: four curricula of ophthalmic education. Am J Ophthalmol. 2007;143(5):859–865. 10.1016/j.ajo.2007.01.055 17452171

[7] Bruce BB, Lamirel C, Wright DW, et al. Nonmydriatic ocular fundus photography in the emergency department. N Engl J Med. 2011;364(4):387–389. 10.1056/NEJMc1009733 21268749 PMC3433395

[8] Albert DM, Bartley GB. A proposal to improve ophthalmic education in medical schools. Ophthalmology. 2014;121(6):1157–1159. 10.1016/j.ophtha.2014.04.003 24893767

[9] Biousse V, Bruce BB, Newman NJ. Ophthalmoscopy in the 21st century: the 2017 H. Houston Merritt Lecture. Neurology. 2018;90(4):167–175. 10.1212/WNL.0000000000004868 29273687 PMC5798658

[10] Bowers EMR, Perzia B, Enzor R, et al. A required ophthalmology rotation: providing medical students with a foundation in eye-related diagnoses and management. MedEdPORTAL. 2021;17:11100. 10.15766/mep_2374-8265.11100 33598541 PMC7880261

[11] Mackay DD, Garza PS, Bruce BB, Newman NJ, Biousse V. The demise of direct ophthalmoscopy: a modern clinical challenge. Neurol Clin Pract. 2015;5(2):150–157. 10.1212/CPJ.0000000000000115 26137422 PMC4404284

[12] Kelly LP, Garza PS, Bruce BB, Graubart EB, Newman NJ, Biousse V. Teaching ophthalmoscopy to medical students (the TOTeMS Study). Am J Ophthalmol. 2013;156(5):1056–1061.E10. 10.1016/j.ajo.2013.06.022 24041982 PMC3805733

[13] Scarpa G, Urban F, Vujosevic S, et al. The nonmydriatic fundus camera in diabetic retinopathy screening: a cost-effective study with evaluation for future large-scale application. J Ophthalmol. 2016;2016:4625096. 10.1155/2016/4625096 27885337 PMC5112304

[14] Pasquel FJ, Hendrick AM, Ryan M, Cason E, Ali MK, Narayan KMV. Cost-effectiveness of different diabetic retinopathy screening modalities. J Diabetes Sci Technol. 2016;10(2):301–307. 10.1177/1932296815624109 PMC4773976 26719134

[15] Teismann N, Neilson J, Keenan J. Quality and feasibility of automated digital retinal imaging in the emergency department. J Emerg Med. 2020;58(1):18–24. 10.1016/j.jemermed.2019.08.034 31718881

[16] Root T. OphthoBook. CreateSpace Independent Publishing Platform; 2009.

[17] Benè KL, Bergus G. When learners become teachers: a review of peer teaching in medical student education. Fam Med. 2014;46(10):783–787. 25646829

[18] Yu TC, Wilson NC, Singh PP, Lemanu DP, Hawken SJ, Hill AG. Medical students-as-teachers: a systematic review of peer-assisted teaching during medical school. Adv Med Educ Pract. 2011;2:157–172. 10.2147/AMEP.S14383 23745087 PMC3661256

[19] Whitmill A, Edwards T, Charles S. Training medical student facilitators of peer-assisted study sessions using an objective standardized teaching exercise. MedEdPORTAL. 2020;16:10898. 10.15766/mep_2374-8265.10898 32656319 PMC7328851

