# Supplementary material for: A Novel Approach to Teaching Fundoscopy Using a Virtual Format
Source: MedEdPORTAL. 2022 May 27;18:11252. doi: 10.15766/mep_2374-8265.11252 (PMC9135915; doi:10.15766/mep_2374-8265.11252)
Supplement: Supplementary file 1 — Pretest.docxSlide Deck.pptxPosttest.docxPostworkshop Handout.pdfMedical Student Session Leader Survey.docx [file mep_2374-8265.11252-s001.zip › A. Pretest.docx]

**APE Intro to Ophthalmology Pre-Test**

This test is for educational purposes only and DOES NOT count towards your grade

Unique Identifier: Favorite color + last 3 digits of cell phone number

Have you had any ophthalmology education prior to this session?

Yes

No

Prior exposure to ophthalmology? (If yes, please briefly explain)

What is your interest level in ophthalmology as a career?

|  | 1 | 2 | 3 | 4 | 5 | 6 | 7 |  |
| --- | --- | --- | --- | --- | --- | --- | --- | --- |
| Minimal |  |  |  |  |  |  |  | Very interested |

How confident are you in your ability to determine if a retina photo is abnormal?

|  | 1 | 2 | 3 | 4 | 5 | 6 | 7 |  |
| --- | --- | --- | --- | --- | --- | --- | --- | --- |
| No confidence |  |  |  |  |  |  |  | Extremely confident |

How confident are you in your ability to recognize a swollen optic nerve?

|  | 1 | 2 | 3 | 4 | 5 | 6 | 7 |  |
| --- | --- | --- | --- | --- | --- | --- | --- | --- |
| No confidence |  |  |  |  |  |  |  | Extremely confident |

How confident are you in your ability to recognize a cupped optic nerve?

|  | 1 | 2 | 3 | 4 | 5 | 6 | 7 |  |
| --- | --- | --- | --- | --- | --- | --- | --- | --- |
| No confidence |  |  |  |  |  |  |  | Extremely confident |

How confident are you in your ability to recognize a pale optic nerve (optic nerve pallor)?

|  | 1 | 2 | 3 | 4 | 5 | 6 | 7 |  |
| --- | --- | --- | --- | --- | --- | --- | --- | --- |
| No confidence |  |  |  |  |  |  |  | Extremely confident |

How confident are you in your ability to recognize a retinal hemorrhage?

|  | 1 | 2 | 3 | 4 | 5 | 6 | 7 |  |
| --- | --- | --- | --- | --- | --- | --- | --- | --- |
| No confidence |  |  |  |  |  |  |  | Extremely confident |

1. What is the main finding in this retinal photo?


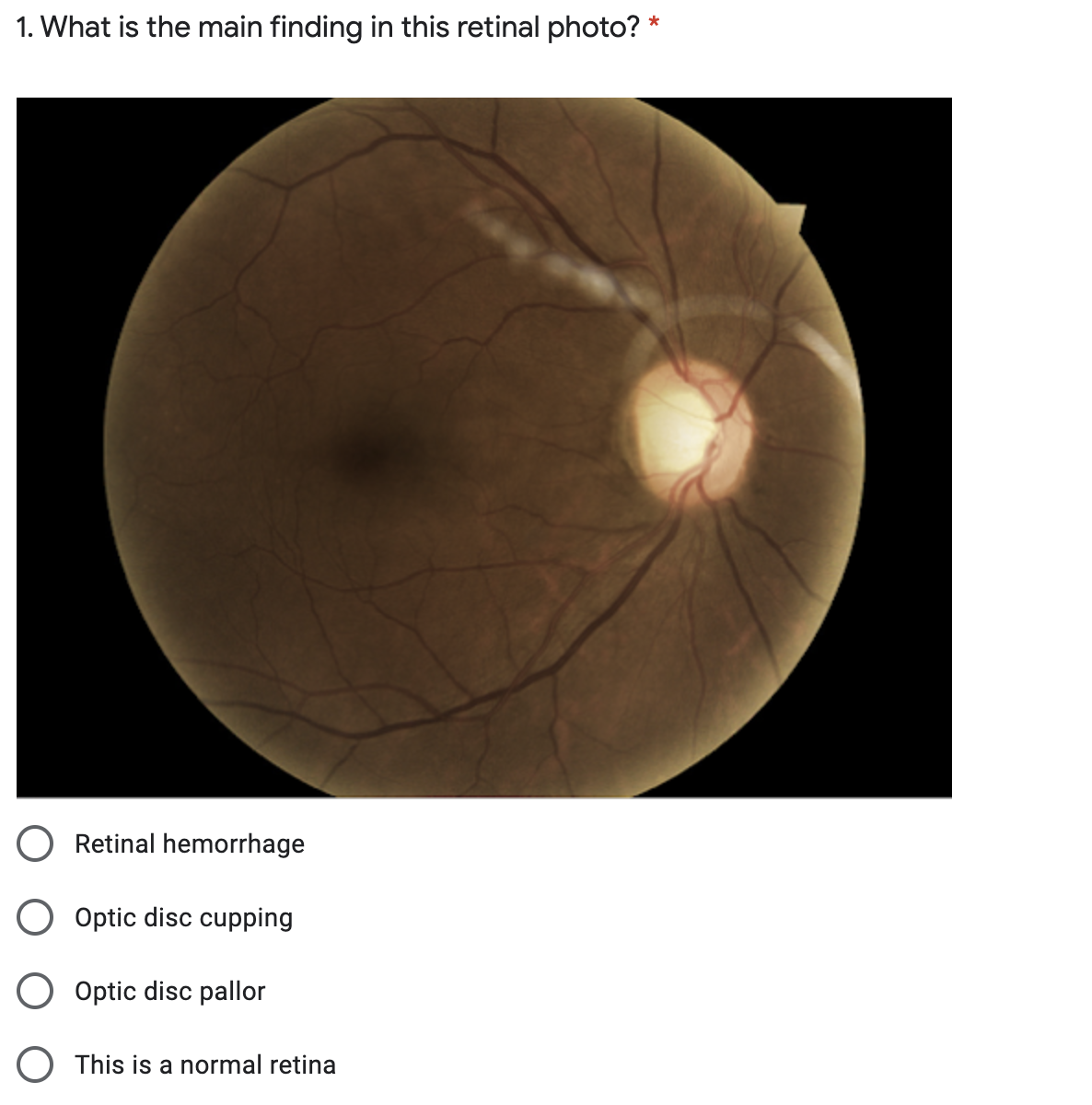


Image is author owned

|  | Retinal hemorrhage |
| --- | --- |
|  | Optic disc cupping |
|  | Optic disc pallor |
|  | This is a normal retina |

1. What is the main finding in this retinal photo?


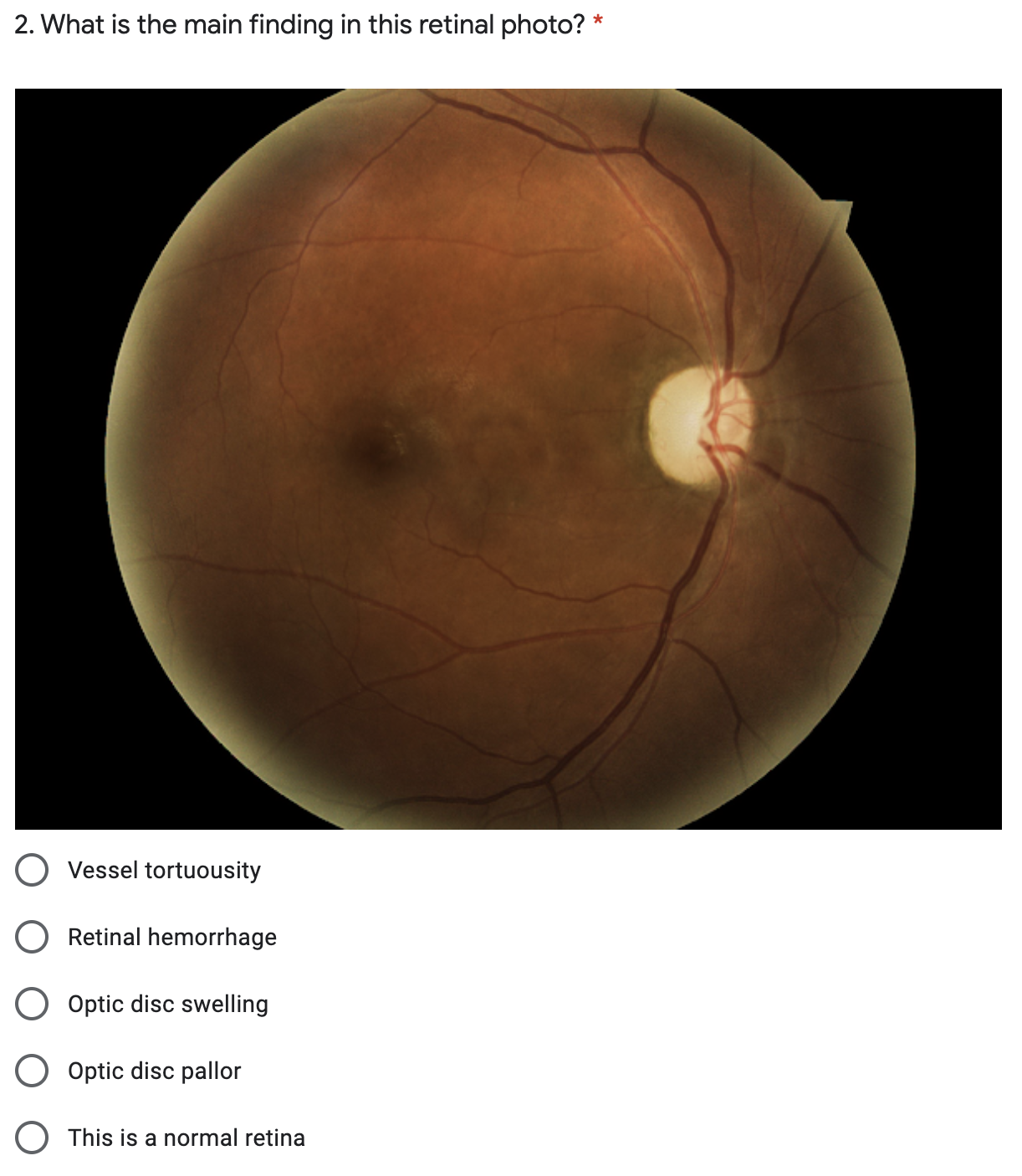


Image is author owned

|  | Vessel tortuosity |
| --- | --- |
|  | Retinal hemorrhage |
|  | Optic disc swelling |
|  | Optic disc pallor |
|  | This is a normal retina |

1. What is the main finding in this retinal photo?


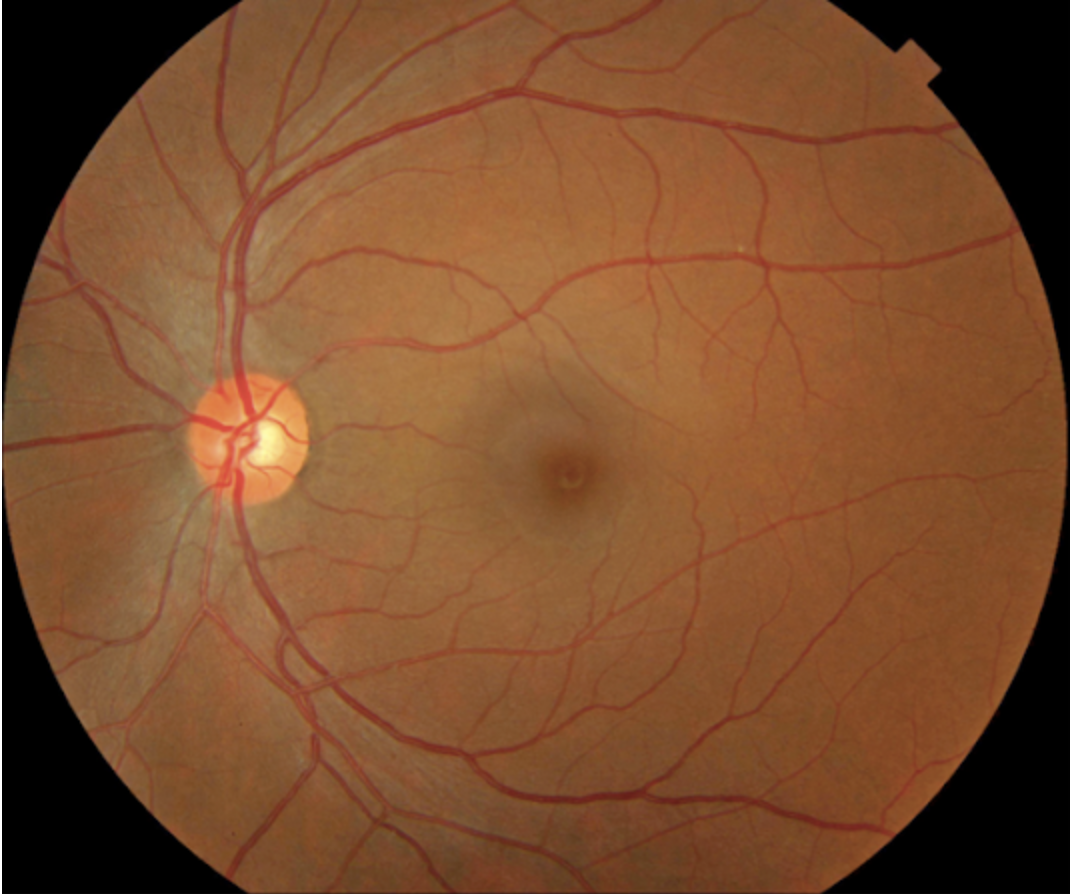


Image is author owned

|  | Retinal hemorrhage |
| --- | --- |
|  | Optic disc swelling |
|  | Optic disc pallor |
|  | Optic disc cupping |
|  | This is a normal retina |

1. Based on the findings in this photograph, what disease is this patient most likely to have?


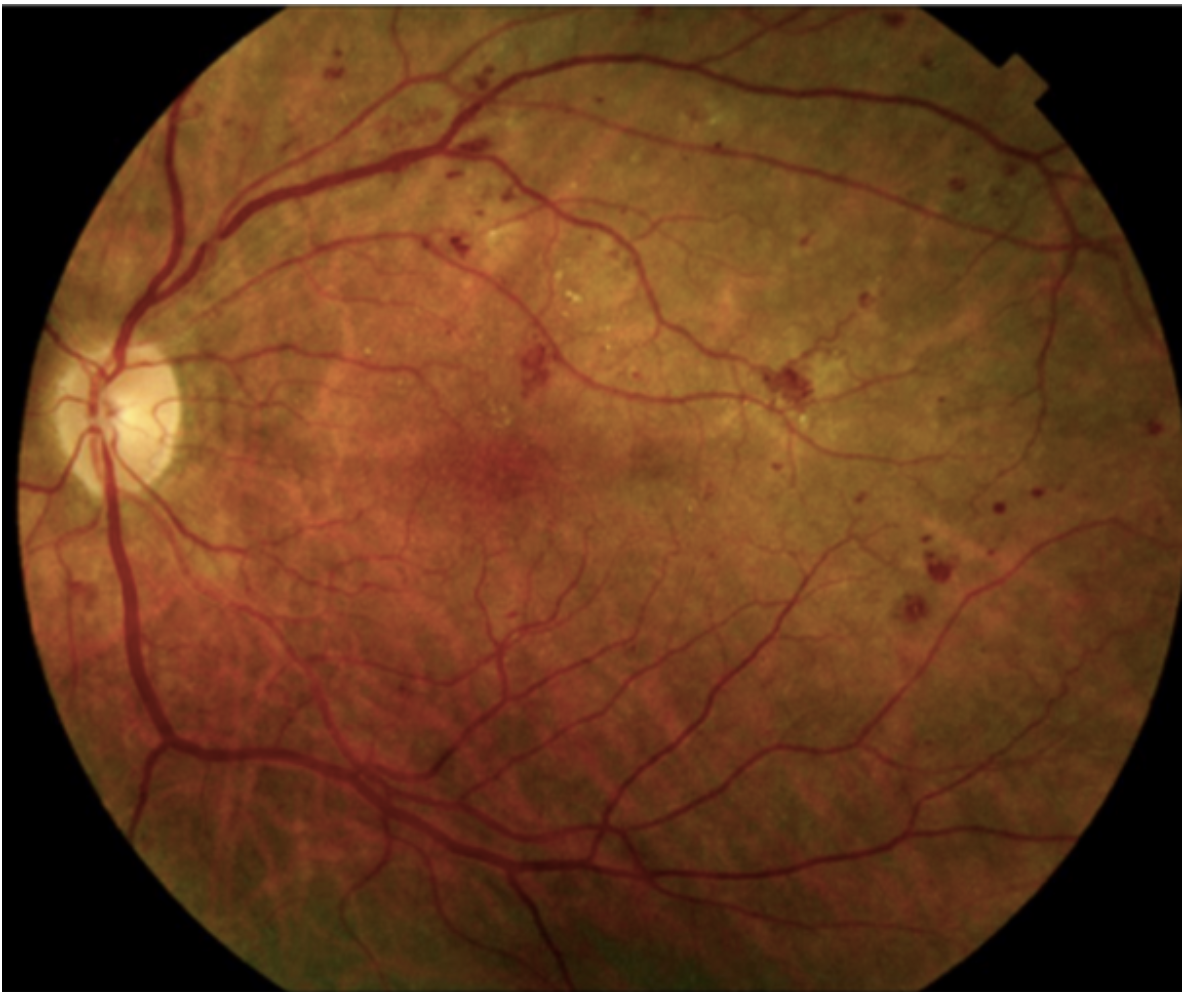


Image is author owned

|  | Glaucoma |
| --- | --- |
|  | Intracranial hypertension |
|  | Diabetes mellitus |
|  | Macular degeneration |

1. Based on the findings in this photograph, what disease is this patient most likely to have?


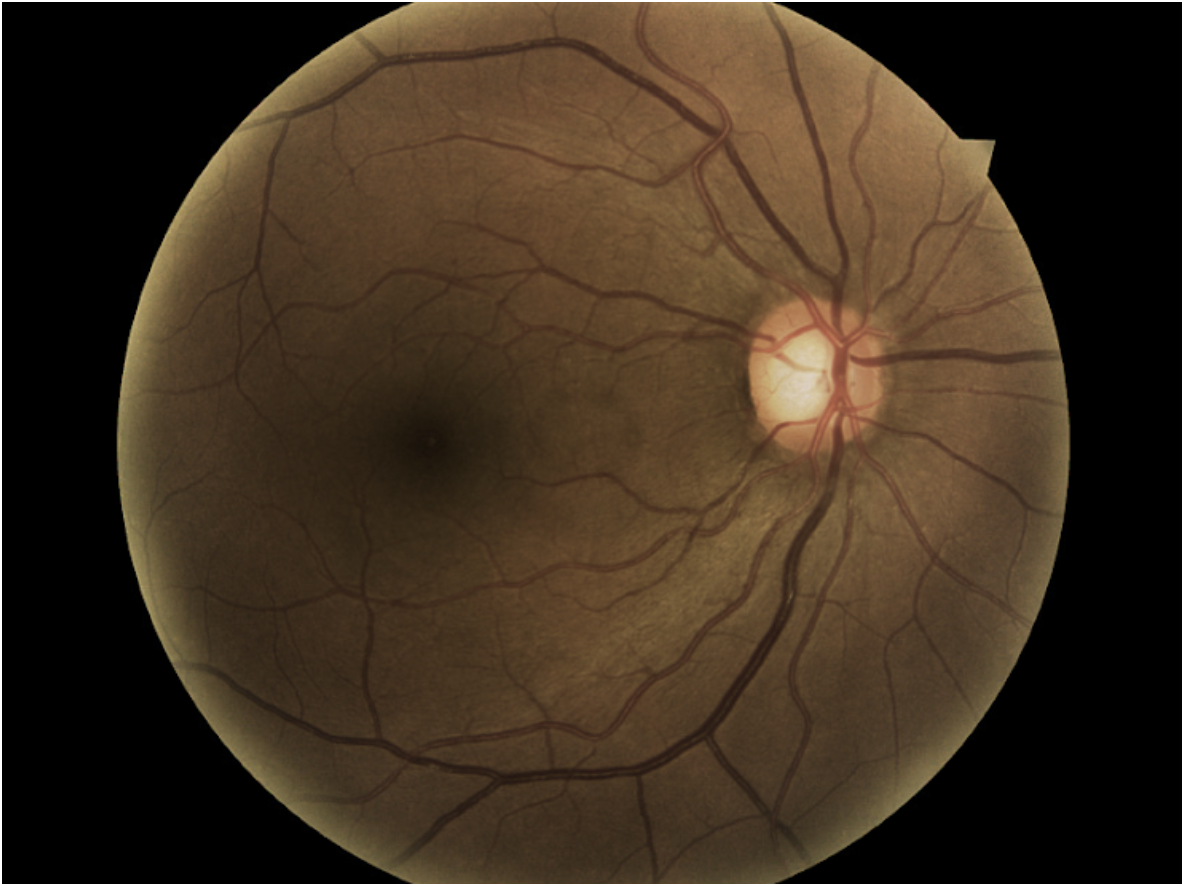


Image is author owned

|  | Glaucoma |
| --- | --- |
|  | Intracranial hypertension |
|  | Diabetes mellitus |
|  | Macular degeneration |

1. What is the main finding in this retinal photo?
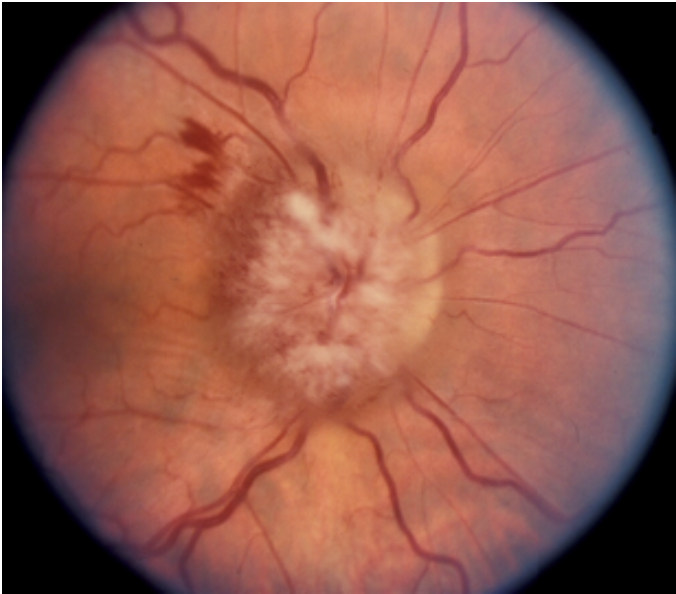


Image is author owned

|  | Normal |
| --- | --- |
|  | Optic disc cupping |
|  | Optic disc swelling |
|  | Optic disc pallor |

1. What is the main finding in this retinal photo?
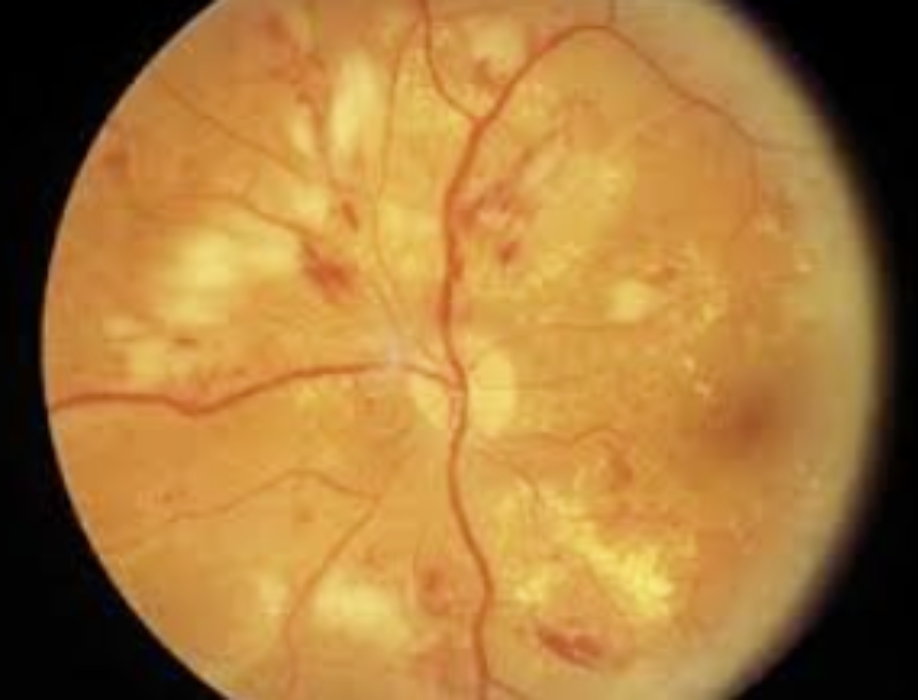


Image is author owned

|  | Cotton wool spots |
| --- | --- |
|  | Optic disc cupping |
|  | Optic disc swelling |
|  | This is a normal retina |

1. What is the main finding in this retinal photo?


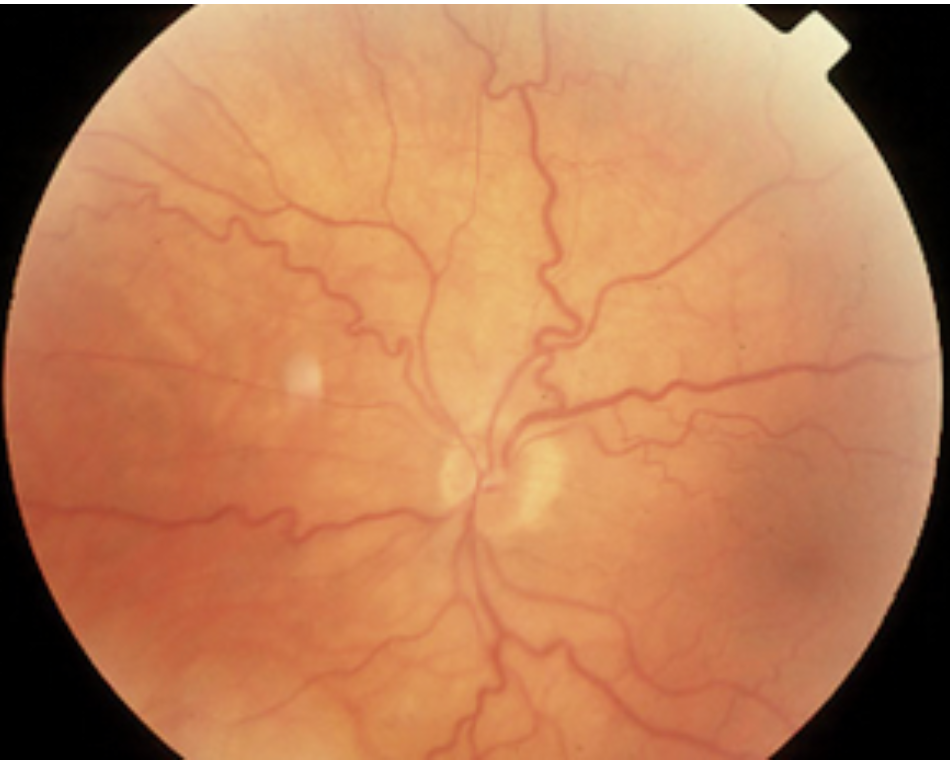


Image is author owned

|  | Neovascularization |
| --- | --- |
|  | AV nicking |
|  | Microaneurysms |
|  | Vessel tortuosity |
|  | This is a normal retina |

1. What is the main finding in this retinal photo?
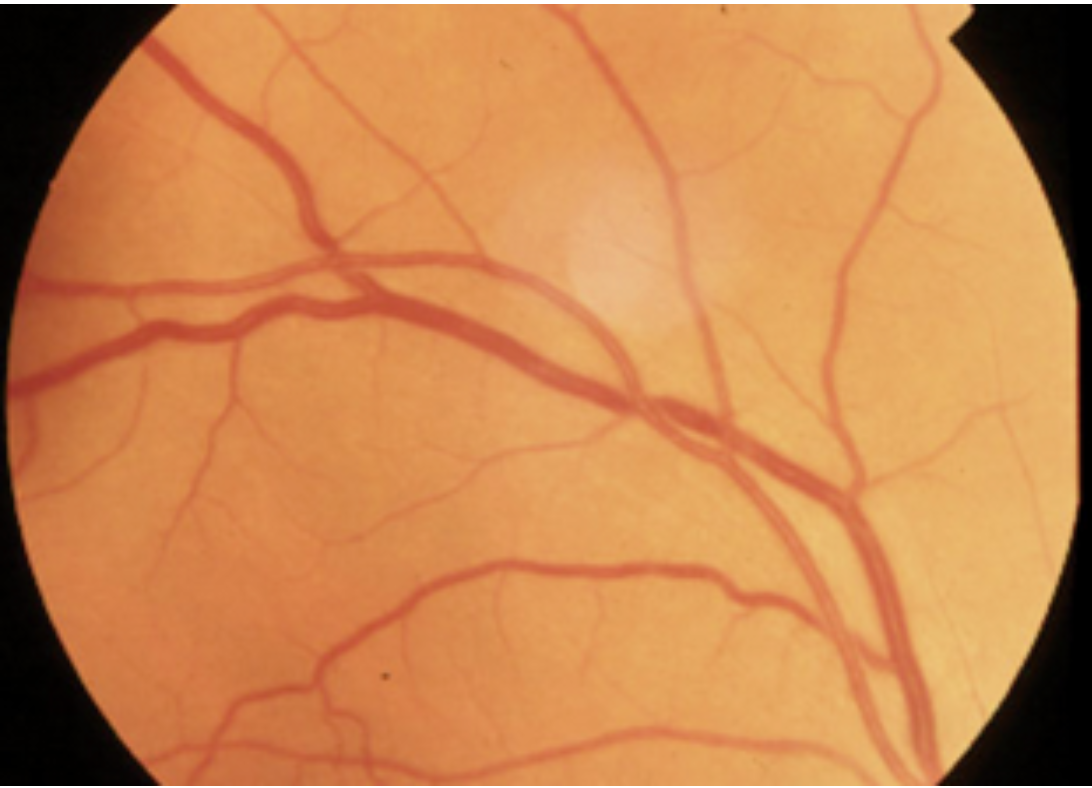


Image is author owned

|  | Retinal neovascularization |
| --- | --- |
|  | AV nicking |
|  | Microaneurysms |
|  | Vessel tortuosity |
|  | This is a normal retina |

1. What is the main finding in this retinal photo?


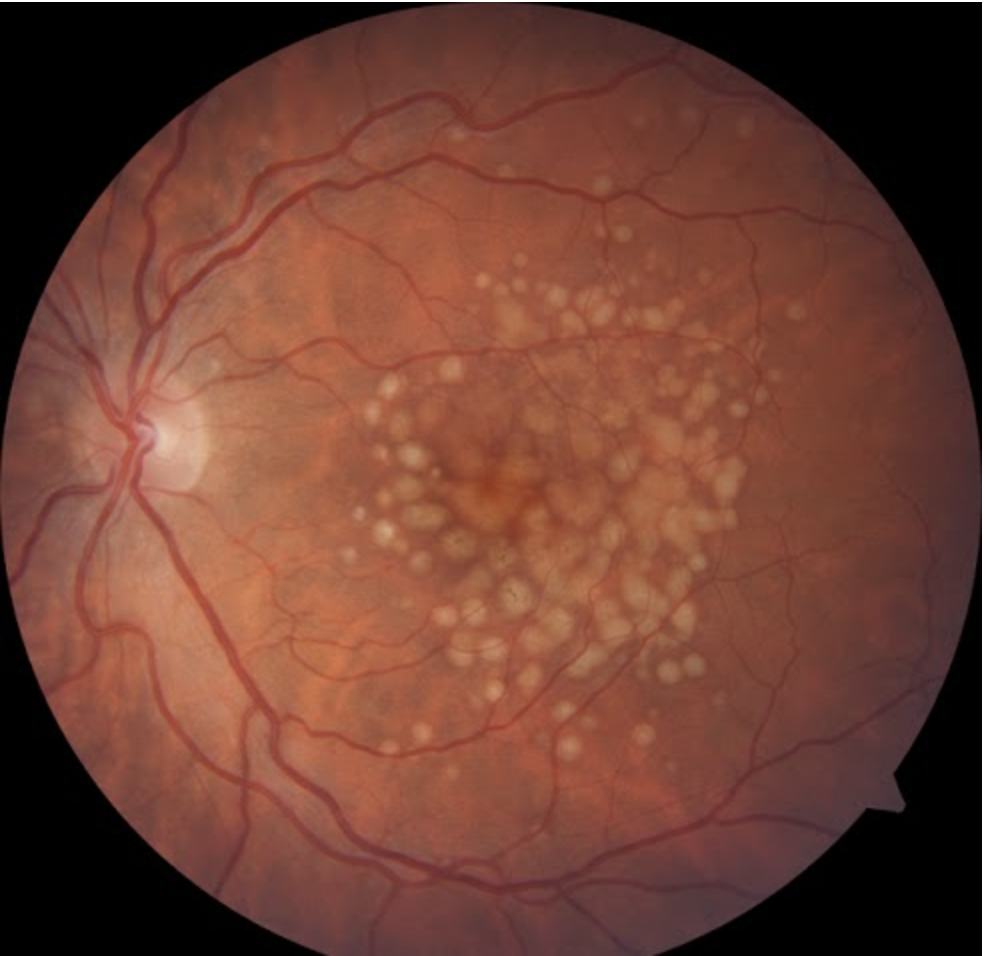


Image is author owned

|  | Vessel nicking |
| --- | --- |
|  | Retinal hemorrhages |
|  | Drusen |
|  | Optic disc cupping |
|  | Cotton wool spots |
|  | This is a normal retina |

1. What is the main finding in this retinal photo?


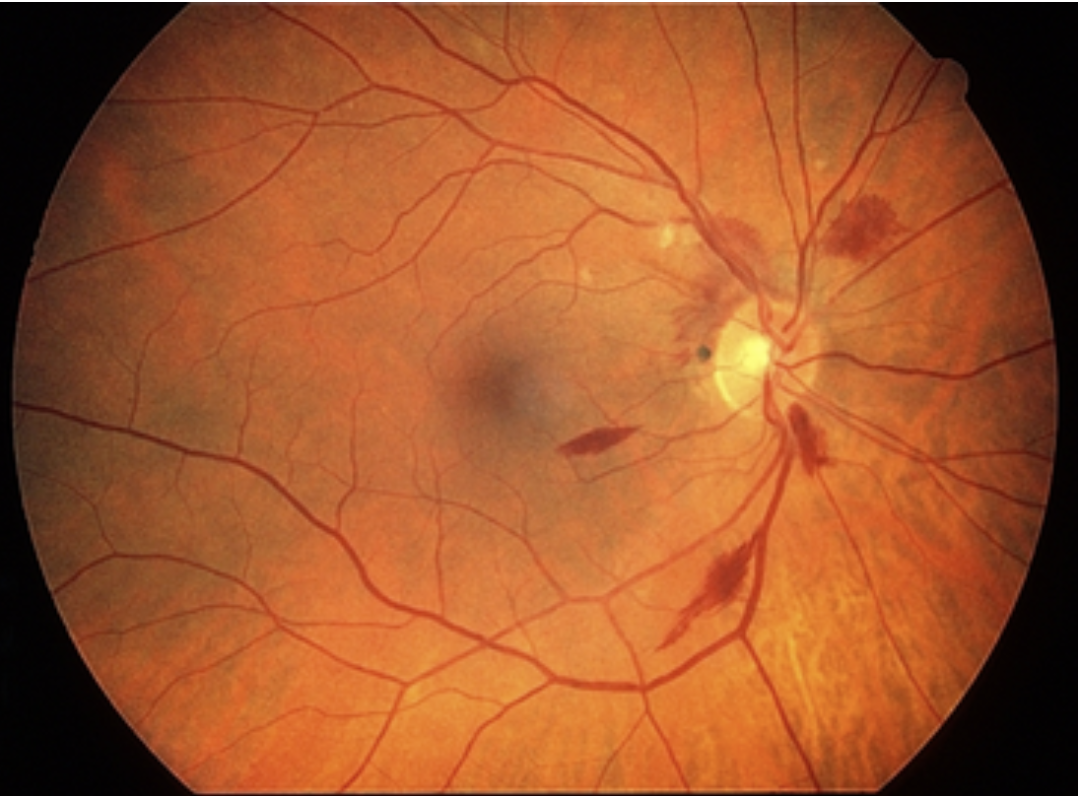


Image is author owned

|  | Flame hemorrhages |
| --- | --- |
|  | Cotton wool spots |
|  | Drusen |
|  | This is a normal retina |

1. You are in the primary care clinic and you take these photos of a patient’s eye. The patient asks you, “what are the white spots scattered in these photos?” Select the most appropriate answer below:


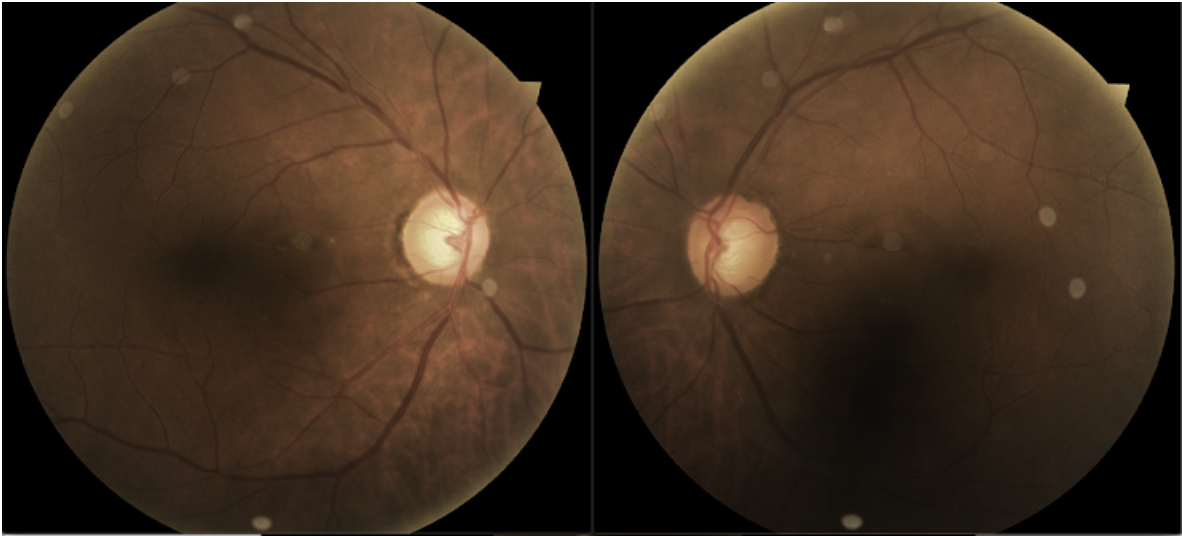


Image is author owned

|  | Cotton wool spots |
| --- | --- |
|  | Dust on camera lens |
|  | Drusen |
|  | Retinal hemorrhages |
|  | Pallor of the optic discs |

1. You are in the primary care clinic and you take this photo of a patient’s eye. Select the most appropriate interpretation of the photo:


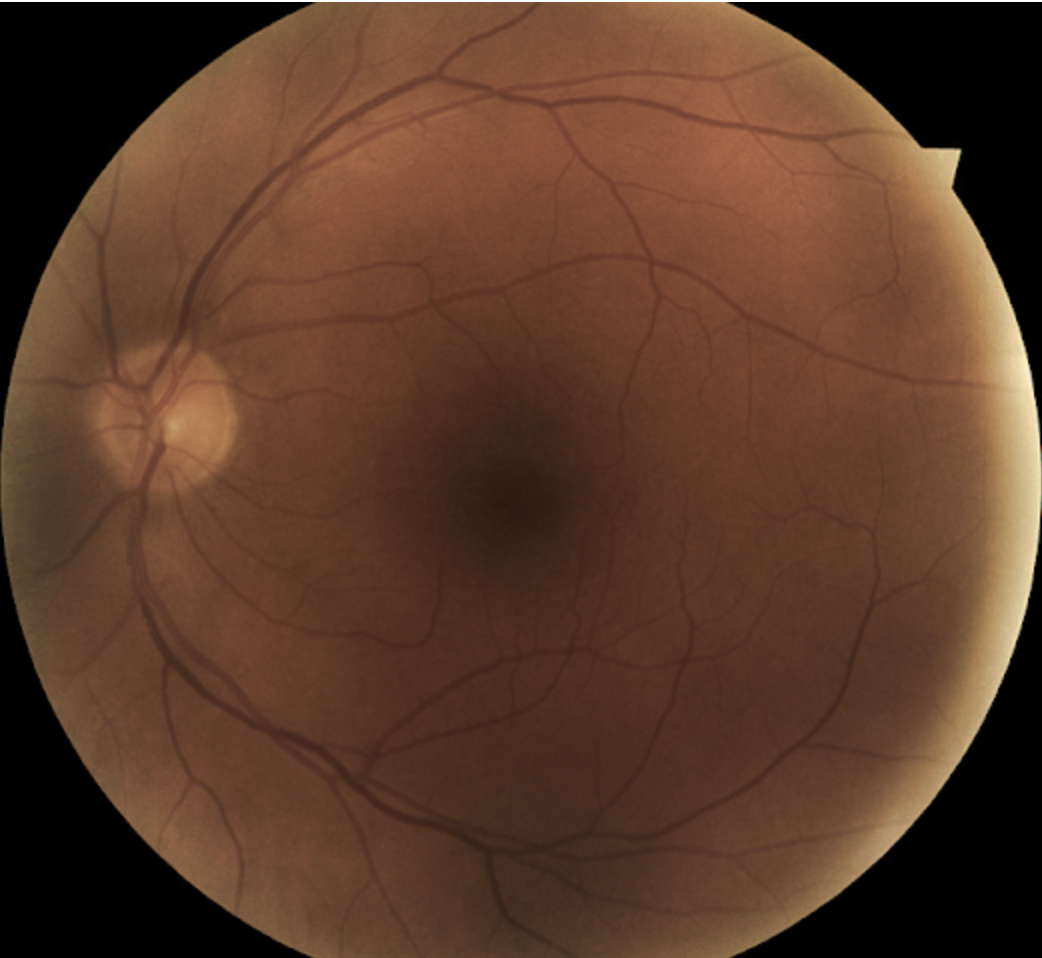


Image is author owned

|  | There is pallor of the optic disc |
| --- | --- |
|  | There is swelling of the optic disc |
|  | There is cupping of the optic disc |
|  | There are retinal hemorrhages |
|  | There are cotton wool spots |
|  | This is a normal retina |

1. A 32-year-old obese woman presents to the emergency department for intermittent blurry vision. You take a retinal photograph (below) and interpret it. What should you do for this patient?


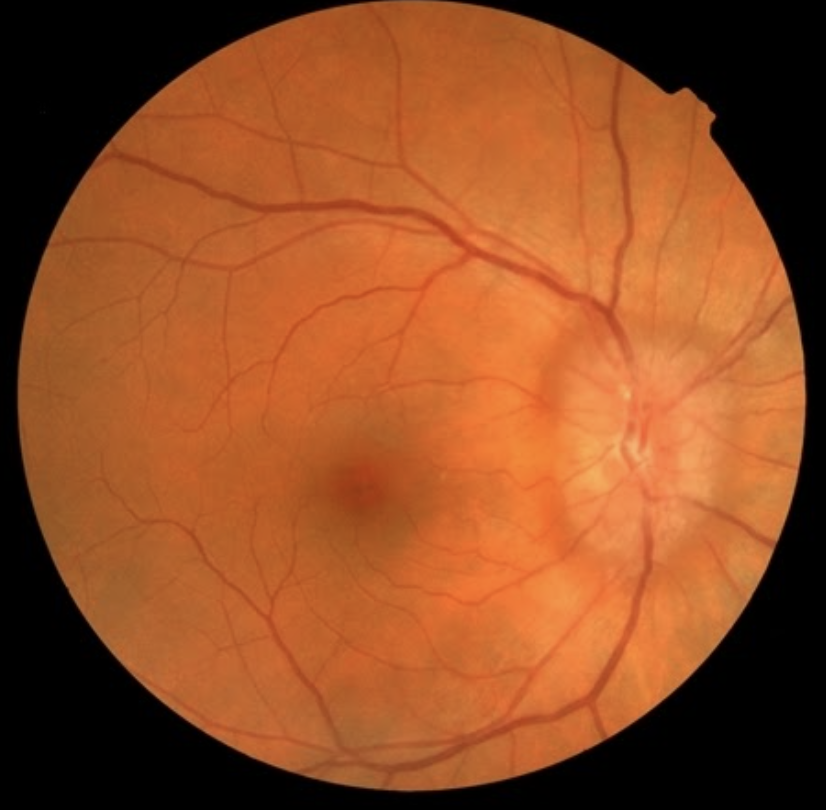


Image is author owned

|  | Check her blood sugar |
| --- | --- |
|  | Measure her intracranial pressure |
|  | Refer her to a glaucoma specialist |
|  | Reassure her that she has a normal eye exam |

1. A 56-year-old man with no past medical history presents to the primary care clinic for a routine health check. You take a retinal photograph (below) and interpret it. Based on your interpretation, what should you do for this inpatient?


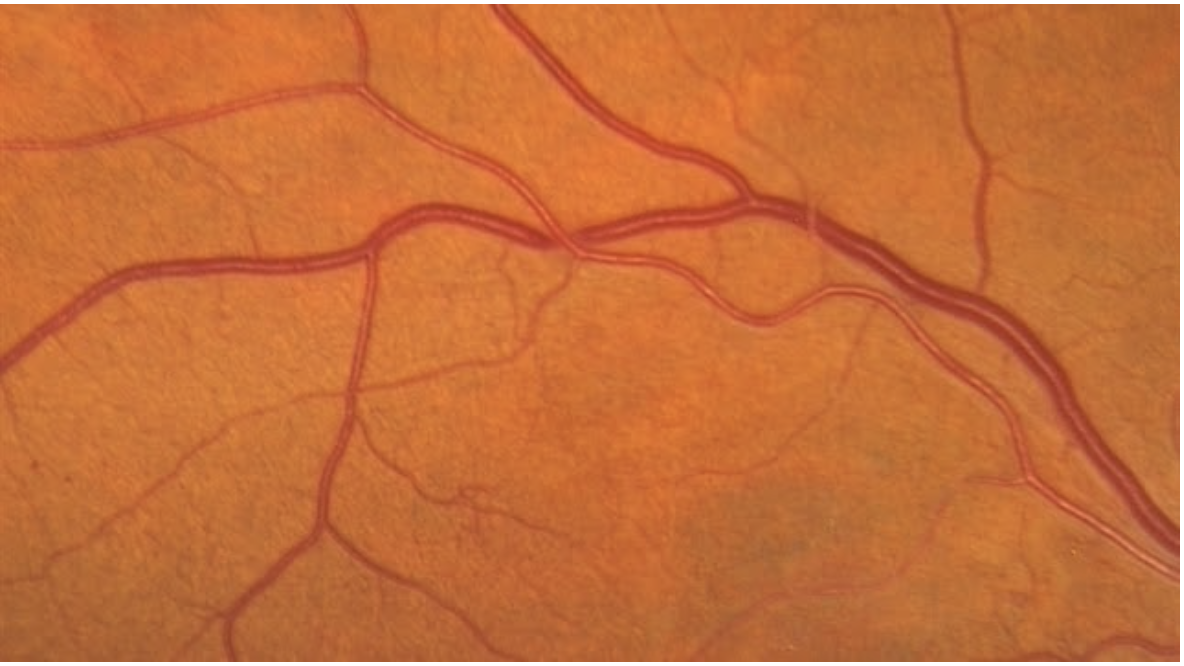


Image is author owned

|  | Check his blood sugar |
| --- | --- |
|  | Check his blood pressure |
|  | Check his intracranial pressure |
|  | Reassure him that he has a normal eye exam |
