# Supplementary material for: A Novel Approach to Teaching Fundoscopy Using a Virtual Format
Source: MedEdPORTAL. 2022 May 27;18:11252. doi: 10.15766/mep_2374-8265.11252 (PMC9135915; doi:10.15766/mep_2374-8265.11252)
Supplement: Supplementary file 1 — Pretest.docxSlide Deck.pptxPosttest.docxPostworkshop Handout.pdfMedical Student Session Leader Survey.docx [file mep_2374-8265.11252-s001.zip › C. Posttest.docx]

**APE Intro to Ophthalmology Post-Test**

This test is for educational purposes only and DOES NOT count towards your grade

Unique Identifier: Favorite color + last 3 digits of cell phone number

Check all that apply – My small group was led by a(n):

|  | Senior medical student |
| --- | --- |
|  | Ophthalmology resident |
|  | Ophthalmology fellow |
|  | Ophthalmology attending |
|  | Other: ________________________________________ |

Prior exposure to ophthalmology? (If yes, please briefly explain)

What is your interest level in ophthalmology as a career?

|  | 1 | 2 | 3 | 4 | 5 | 6 | 7 |  |
| --- | --- | --- | --- | --- | --- | --- | --- | --- |
| Minimal |  |  |  |  |  |  |  | Very interested |

How confident are you in your ability to determine if a retina photo is abnormal?

|  | 1 | 2 | 3 | 4 | 5 | 6 | 7 |  |
| --- | --- | --- | --- | --- | --- | --- | --- | --- |
| No confidence |  |  |  |  |  |  |  | Extremely confident |

How confident are you in your ability to recognize a swollen optic nerve?

|  | 1 | 2 | 3 | 4 | 5 | 6 | 7 |  |
| --- | --- | --- | --- | --- | --- | --- | --- | --- |
| No confidence |  |  |  |  |  |  |  | Extremely confident |

How confident are you in your ability to recognize a cupped optic nerve?

|  | 1 | 2 | 3 | 4 | 5 | 6 | 7 |  |
| --- | --- | --- | --- | --- | --- | --- | --- | --- |
| No confidence |  |  |  |  |  |  |  | Extremely confident |

How confident are you in your ability to recognize a pale optic nerve (optic nerve pallor)?

|  | 1 | 2 | 3 | 4 | 5 | 6 | 7 |  |
| --- | --- | --- | --- | --- | --- | --- | --- | --- |
| No confidence |  |  |  |  |  |  |  | Extremely confident |

How confident are you in your ability to recognize a retinal hemorrhage?

|  | 1 | 2 | 3 | 4 | 5 | 6 | 7 |  |
| --- | --- | --- | --- | --- | --- | --- | --- | --- |
| No confidence |  |  |  |  |  |  |  | Extremely confident |

1. What is the main finding in this retinal photo?


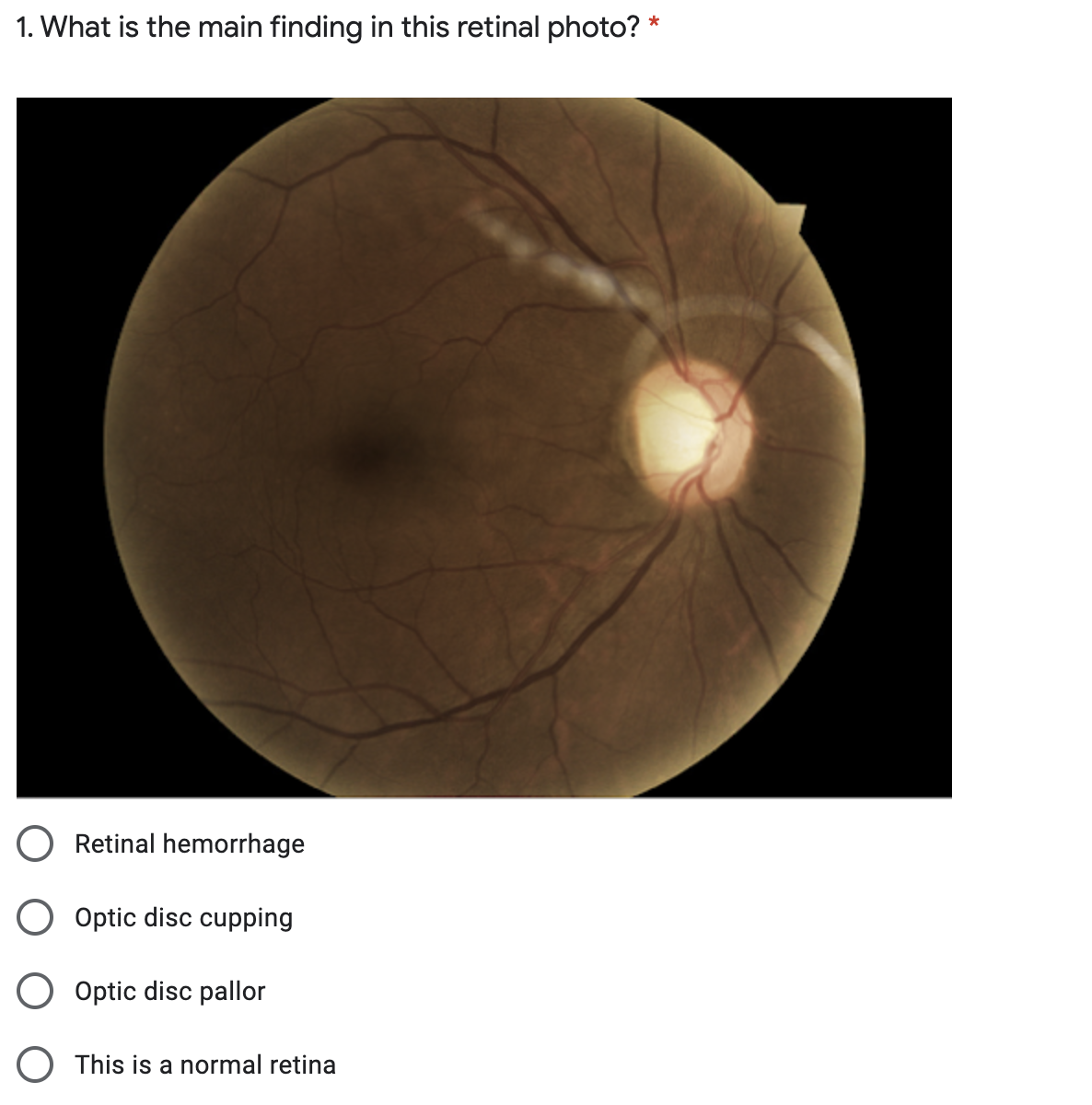


Image is author owned

|  | Retinal hemorrhage |
| --- | --- |
|  | Optic disc cupping |
|  | Optic disc pallor |
|  | This is a normal retina |

1. What is the main finding in this retinal photo?


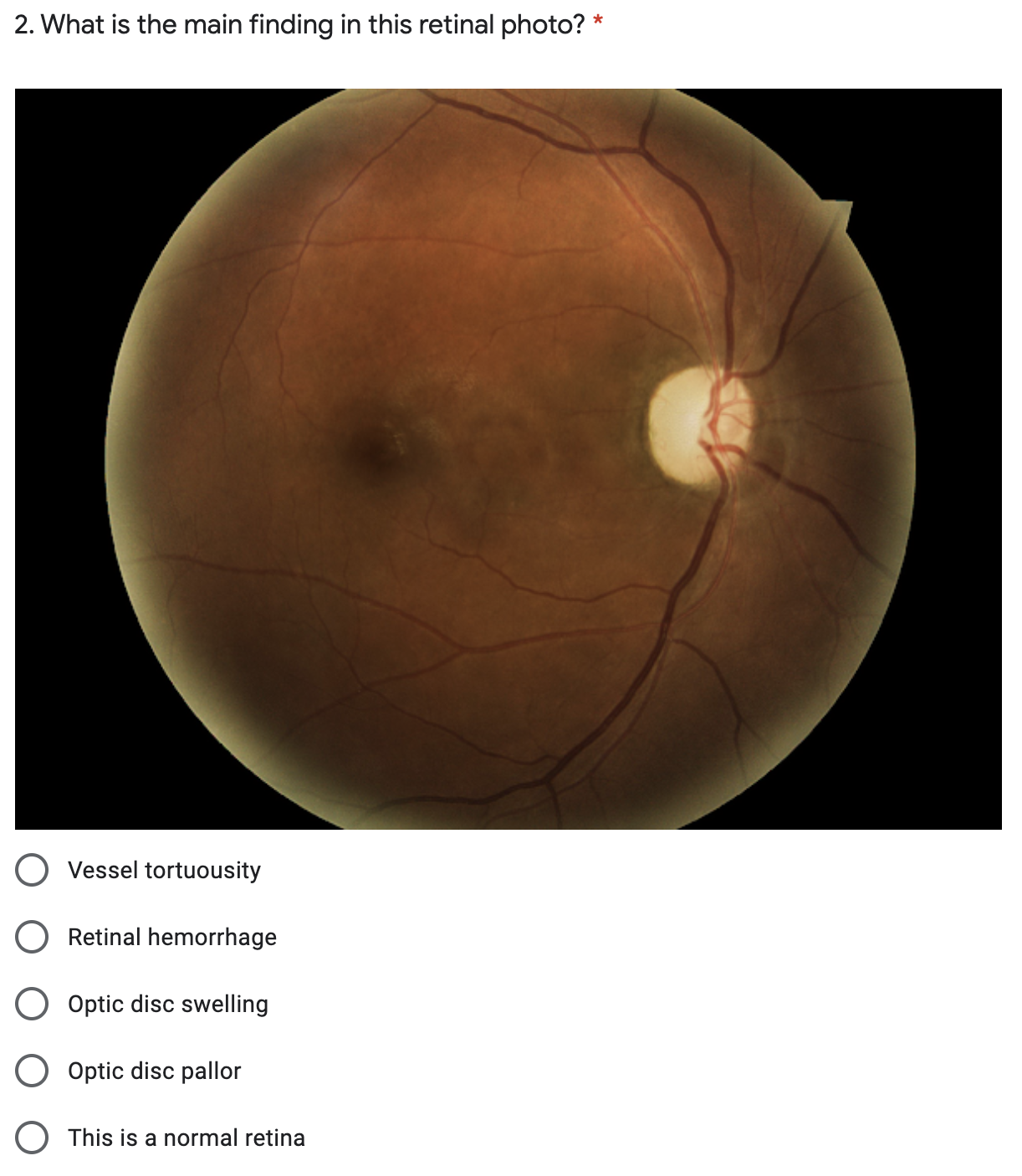


Image is author owned

|  | Vessel tortuosity |
| --- | --- |
|  | Retinal hemorrhage |
|  | Optic disc swelling |
|  | Optic disc pallor |
|  | This is a normal retina |

1. What is the main finding in this retinal photo?


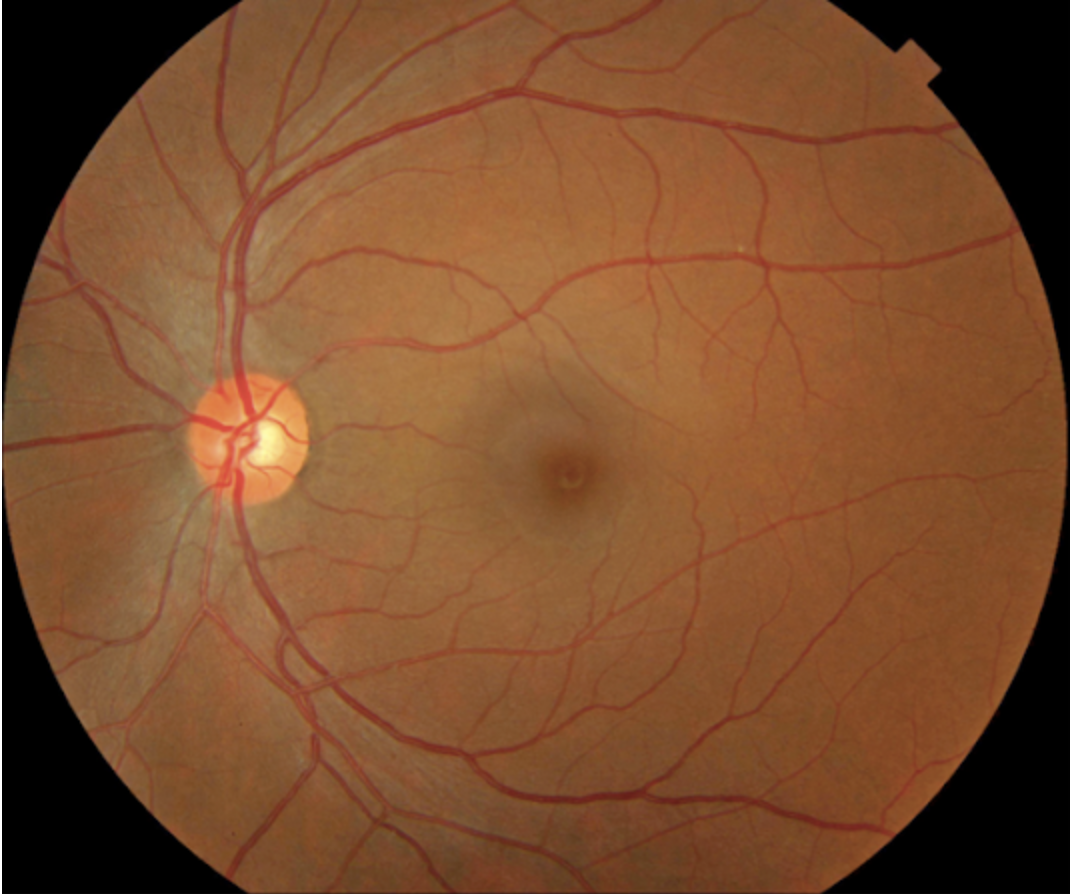


Image is author owned

|  | Retinal hemorrhage |
| --- | --- |
|  | Optic disc swelling |
|  | Optic disc pallor |
|  | Optic disc cupping |
|  | This is a normal retina |

1. Based on the findings in this photograph, what disease is this patient most likely to have?


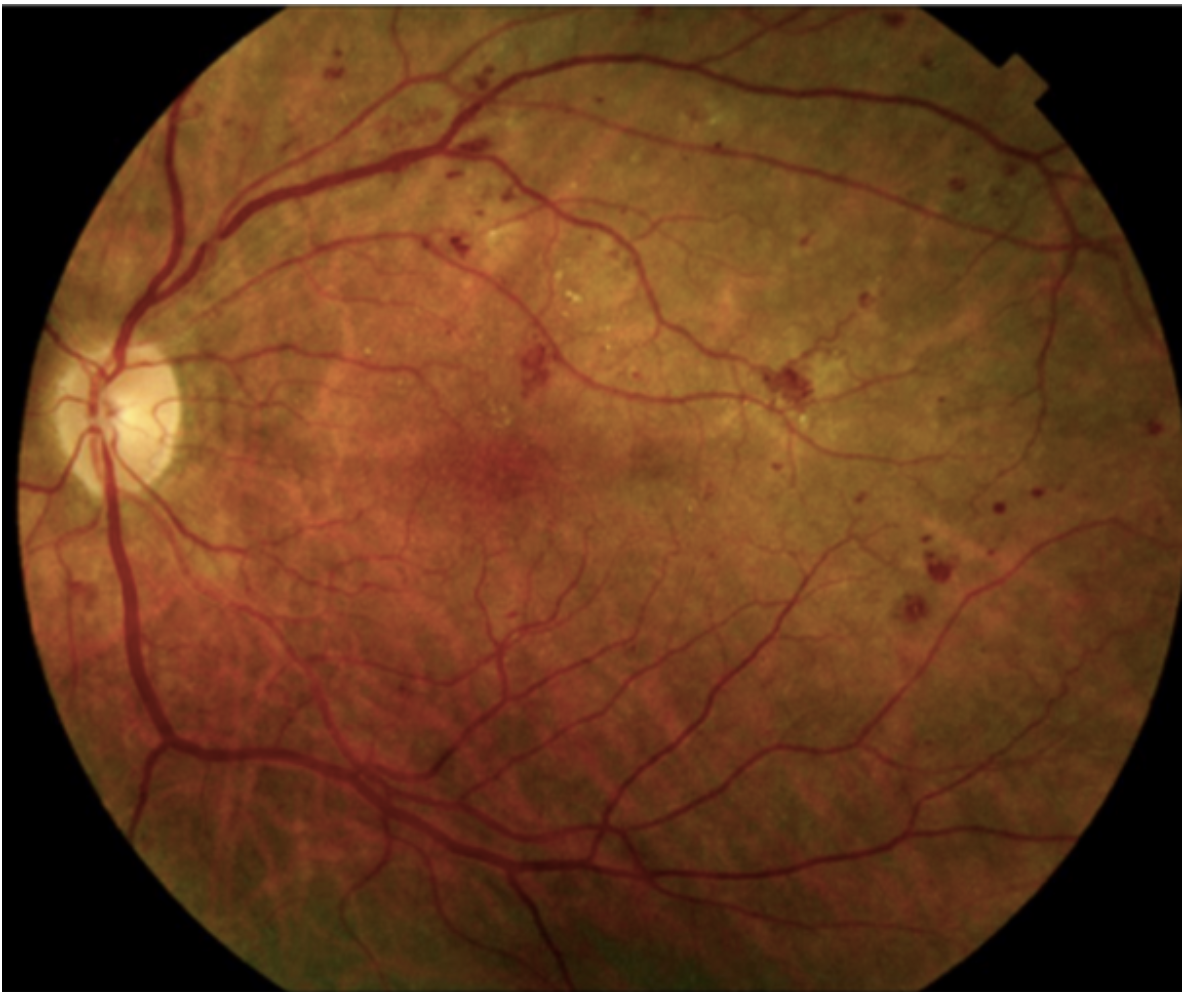


Image is author owned

|  | Glaucoma |
| --- | --- |
|  | Intracranial hypertension |
|  | Diabetes mellitus |
|  | Macular degeneration |

1. Based on the findings in this photograph, what disease is this patient most likely to have?


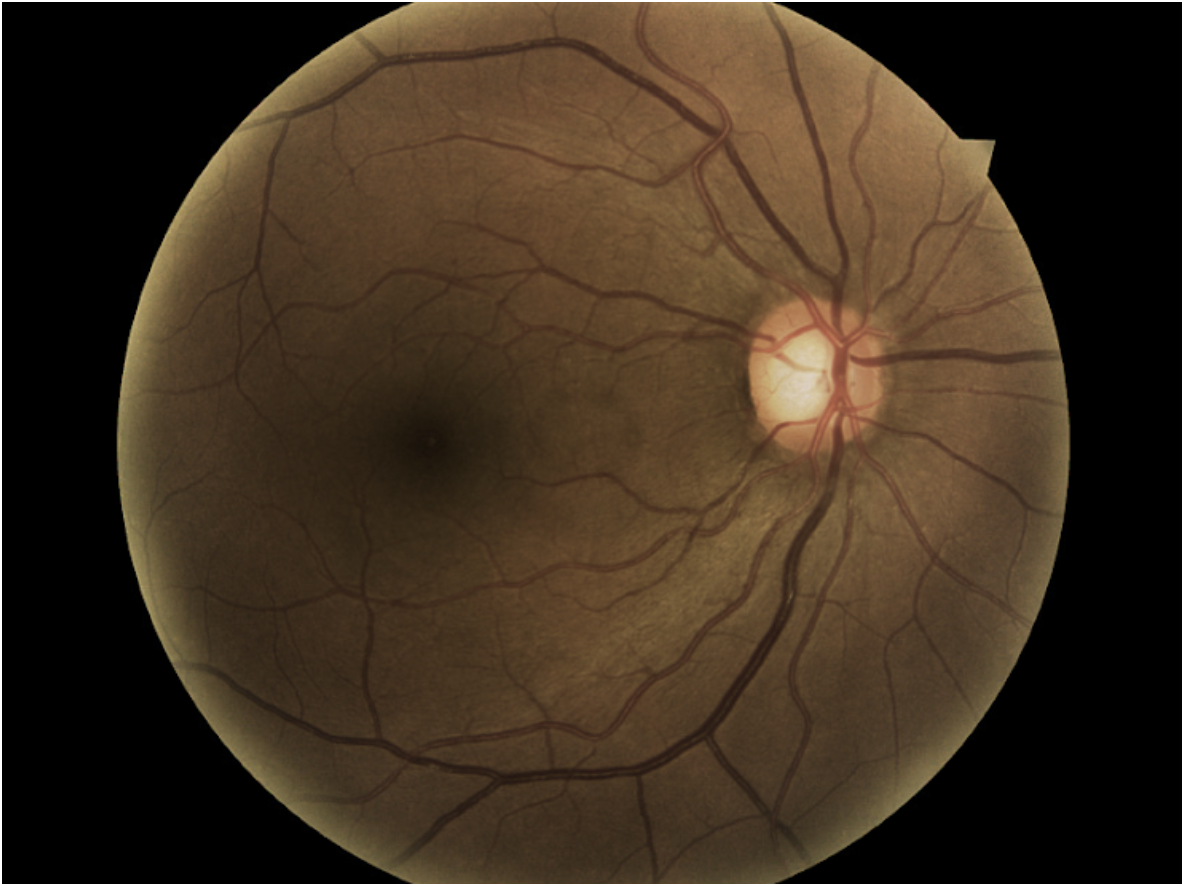


Image is author owned

|  | Glaucoma |
| --- | --- |
|  | Intracranial hypertension |
|  | Diabetes mellitus |
|  | Macular degeneration |

1. What is the main finding in this retinal photo?
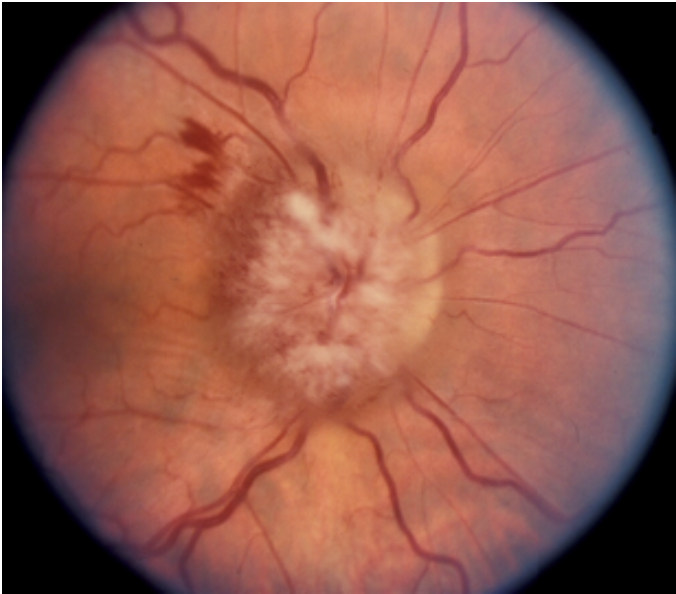


Image is author owned

|  | Normal |
| --- | --- |
|  | Optic disc cupping |
|  | Optic disc swelling |
|  | Optic disc pallor |

1. What is the main finding in this retinal photo?
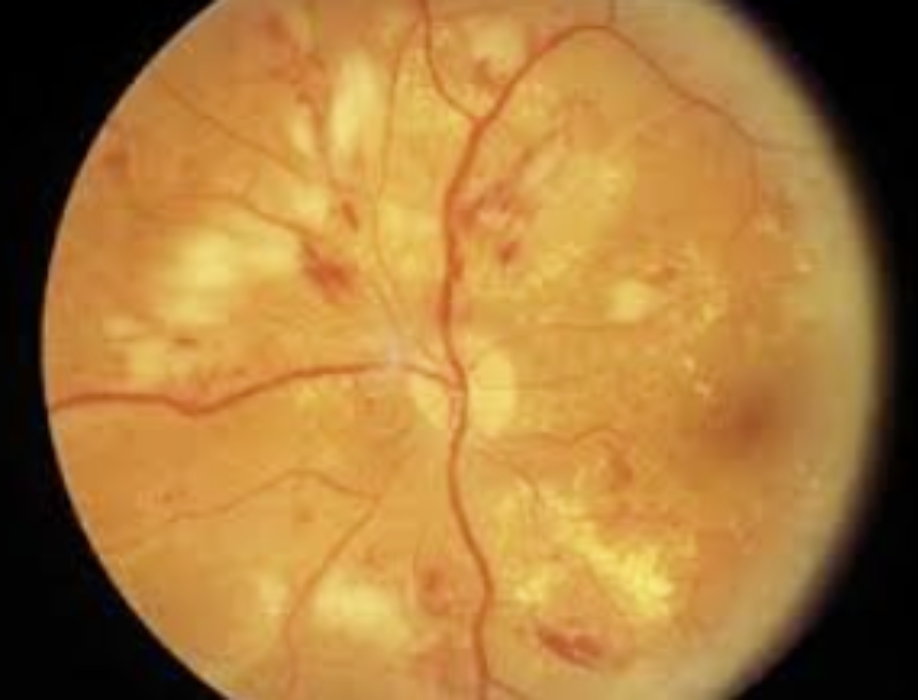


Image is author owned

|  | Cotton wool spots |
| --- | --- |
|  | Optic disc cupping |
|  | Optic disc swelling |
|  | This is a normal retina |

1. What is the main finding in this retinal photo?


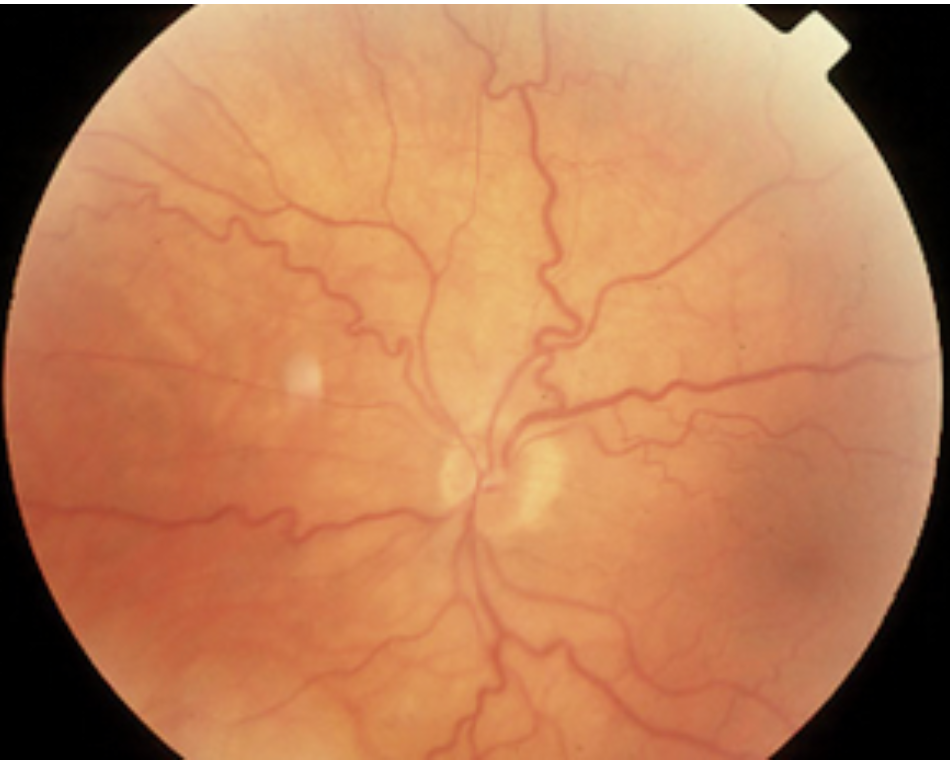


Image is author owned

|  | Neovascularization |
| --- | --- |
|  | AV nicking |
|  | Microaneurysms |
|  | Vessel tortuosity |
|  | This is a normal retina |

1. What is the main finding in this retinal photo?
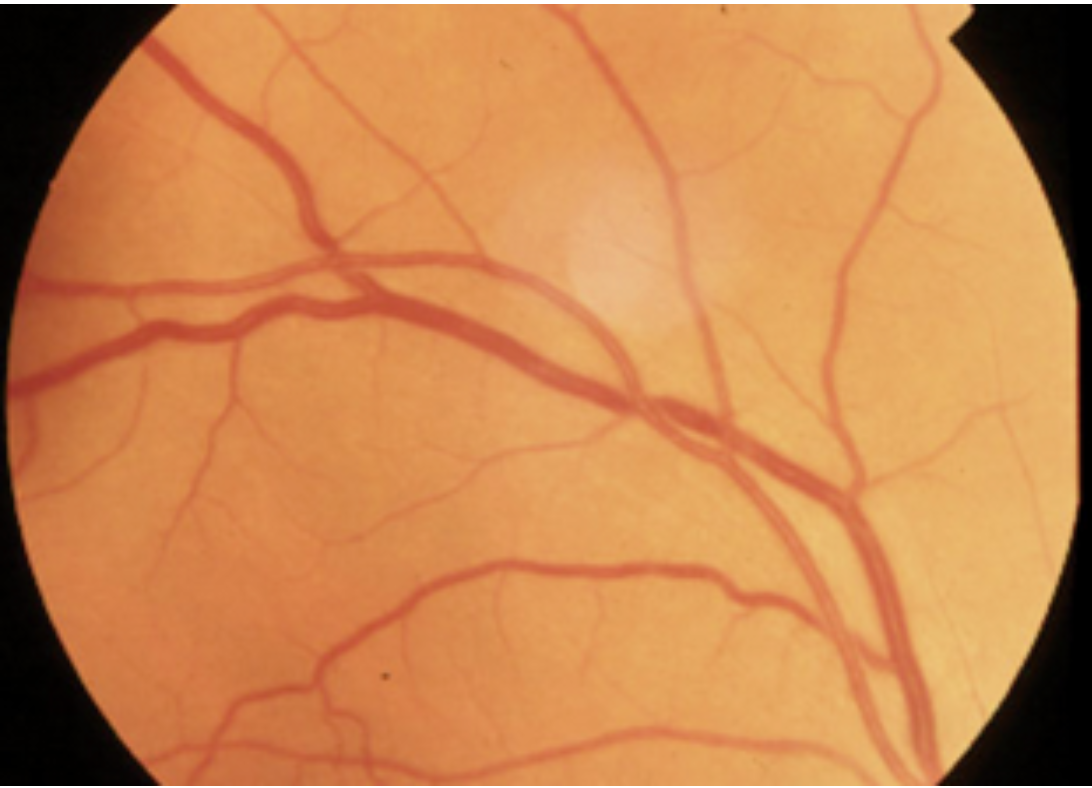


Image is author owned

|  | Retinal neovascularization |
| --- | --- |
|  | AV nicking |
|  | Microaneurysms |
|  | Vessel tortuosity |
|  | This is a normal retina |

1. What is the main finding in this retinal photo?


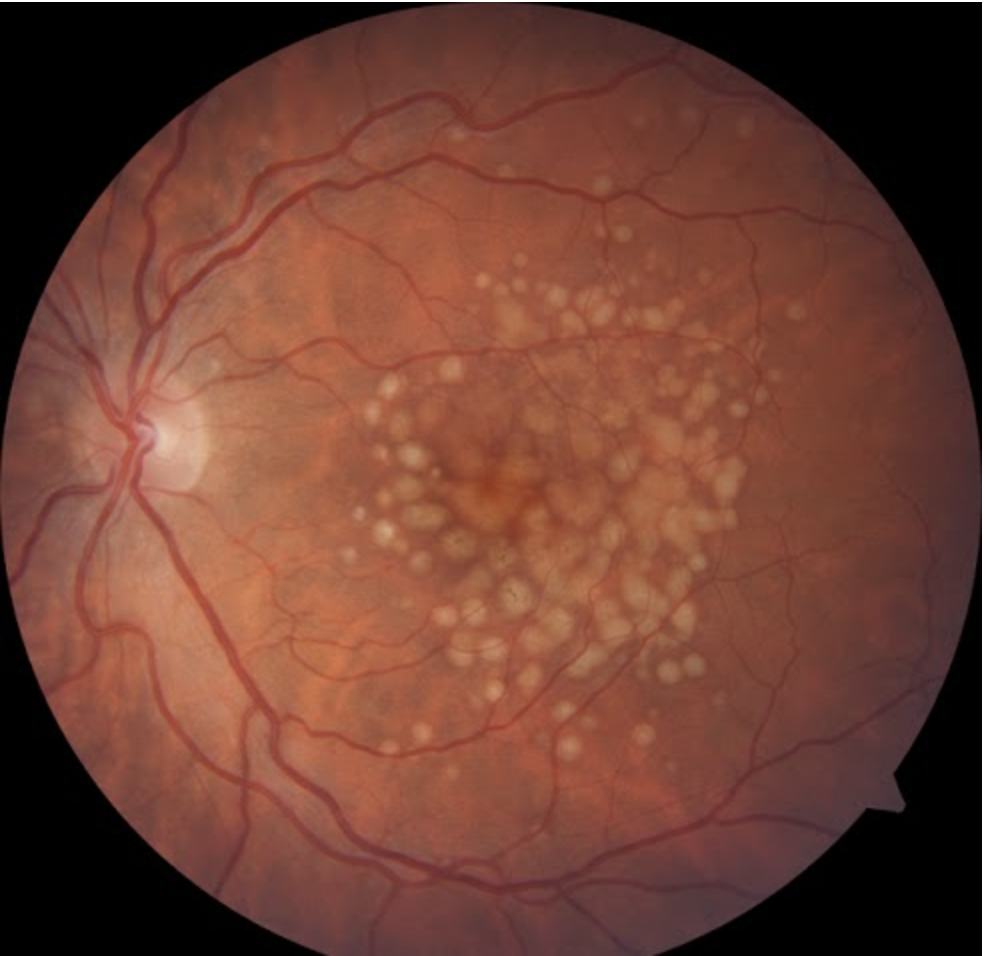


Image is author owned

|  | Vessel nicking |
| --- | --- |
|  | Retinal hemorrhages |
|  | Drusen |
|  | Optic disc cupping |
|  | Cotton wool spots |
|  | This is a normal retina |

1. What is the main finding in this retinal photo?


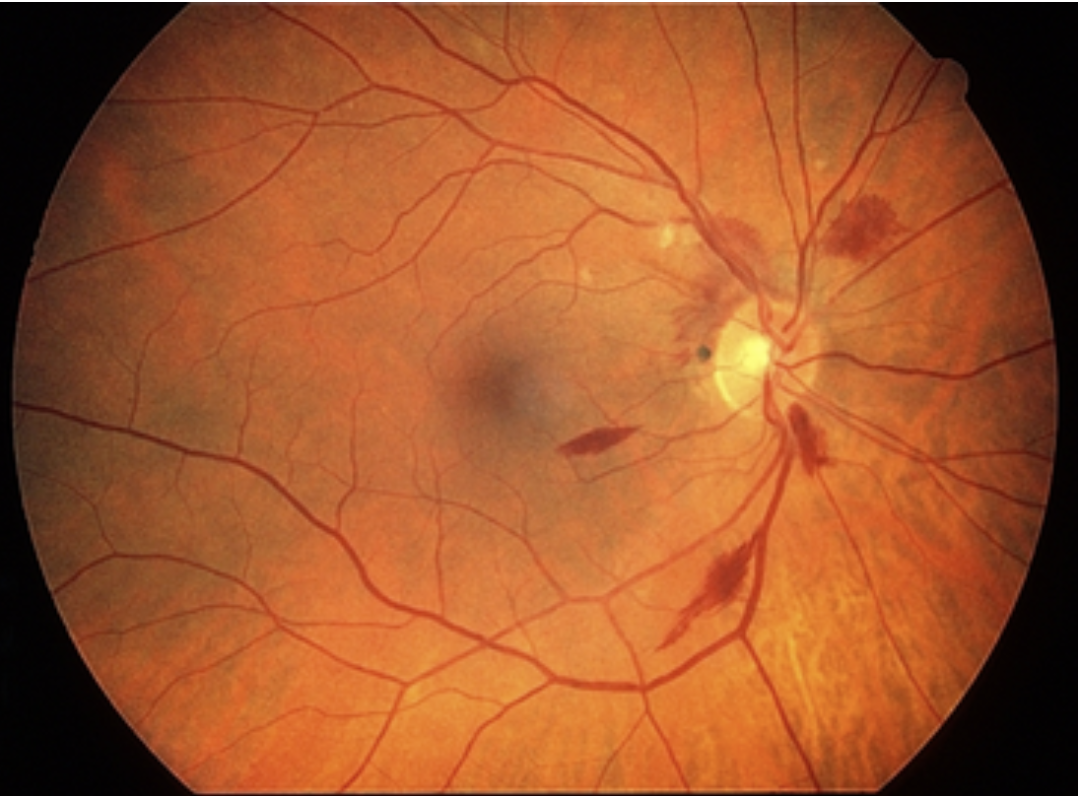


Image is author owned

|  | Flame hemorrhages |
| --- | --- |
|  | Cotton wool spots |
|  | Drusen |
|  | This is a normal retina |

1. You are in the primary care clinic and you take these photos of a patient’s eye. The patient asks you, “what are the white spots scattered in these photos?” Select the most appropriate answer below:


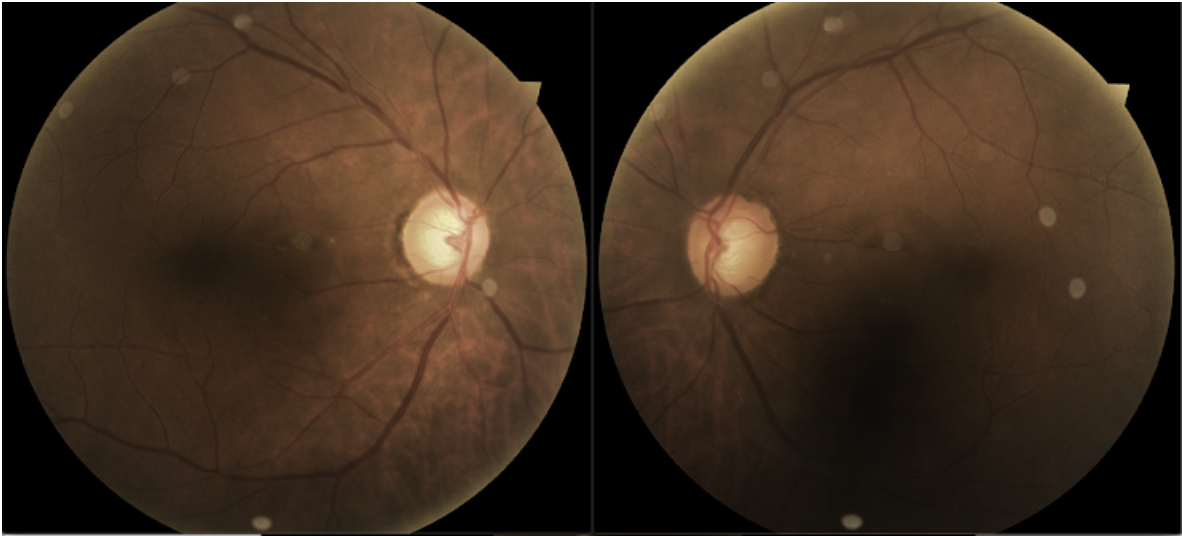


Image is author owned

|  | Cotton wool spots |
| --- | --- |
|  | Dust on cameral lens |
|  | Drusen |
|  | Retinal hemorrhages |
|  | Pallor of the optic discs |

1. You are in the primary care clinic and you take this photo of a patient’s eye. Select the most appropriate interpretation of the photo:


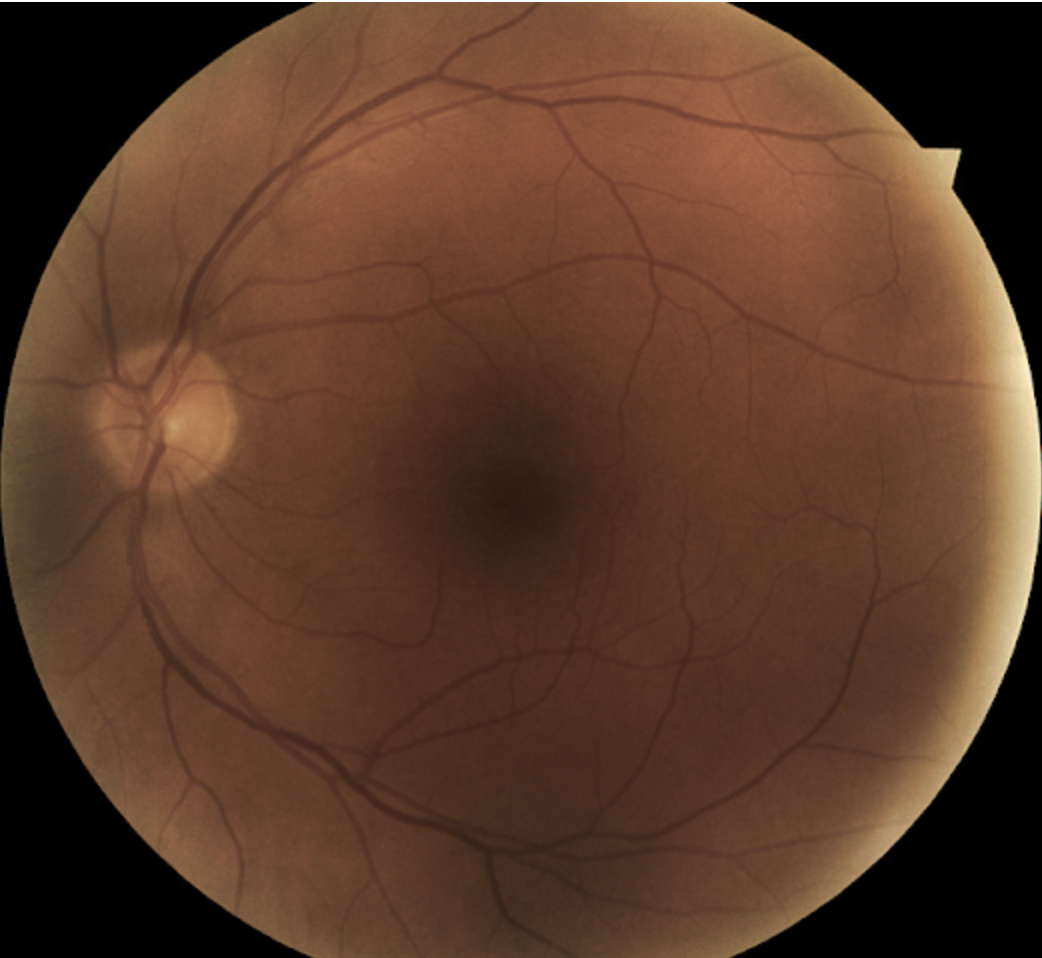
 Image is author owned

|  | There is pallor of the optic disc |
| --- | --- |
|  | There is swelling of the optic disc |
|  | There is cupping of the optic disc |
|  | There are retinal hemorrhages |
|  | There are cotton wool spots |
|  | This is a normal retina |

1. A 32-year-old obese woman presents to the emergency department for intermittent blurry vision. You take a retinal photograph (below) and interpret it. What should you do for this patient?


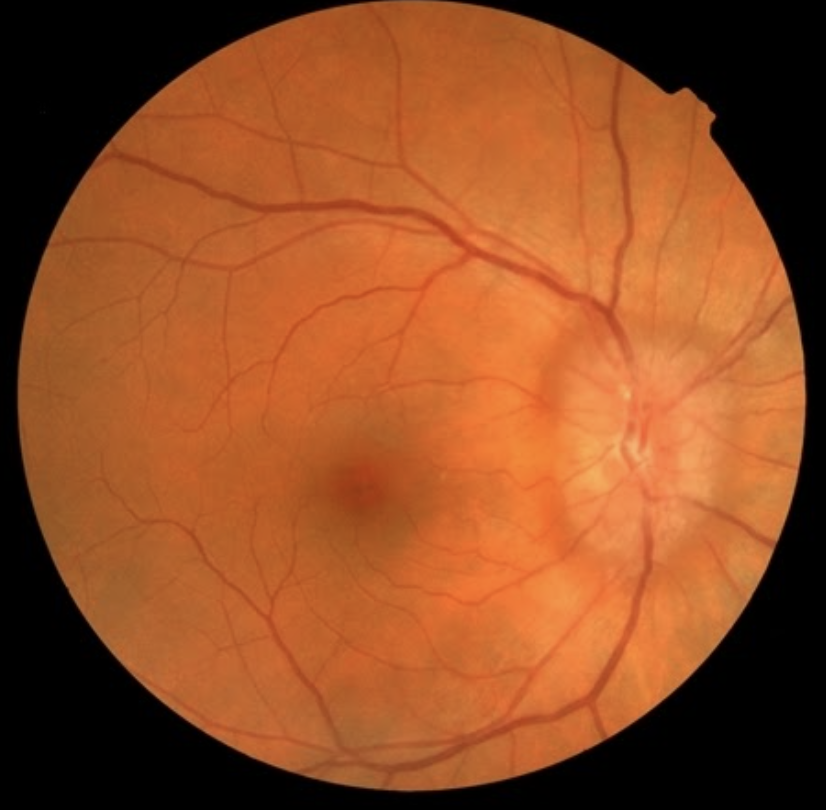


Image is author owned

|  | Check her blood sugar |
| --- | --- |
|  | Measure her intracranial pressure |
|  | Refer her to a glaucoma specialist |
|  | Reassure her that she has a normal eye exam |

1. A 56-year-old man with no past medical history presents to the primary care clinic for a routine health check. You take a retinal photograph (below) and interpret it. Based on your interpretation, what should you do for this inpatient?


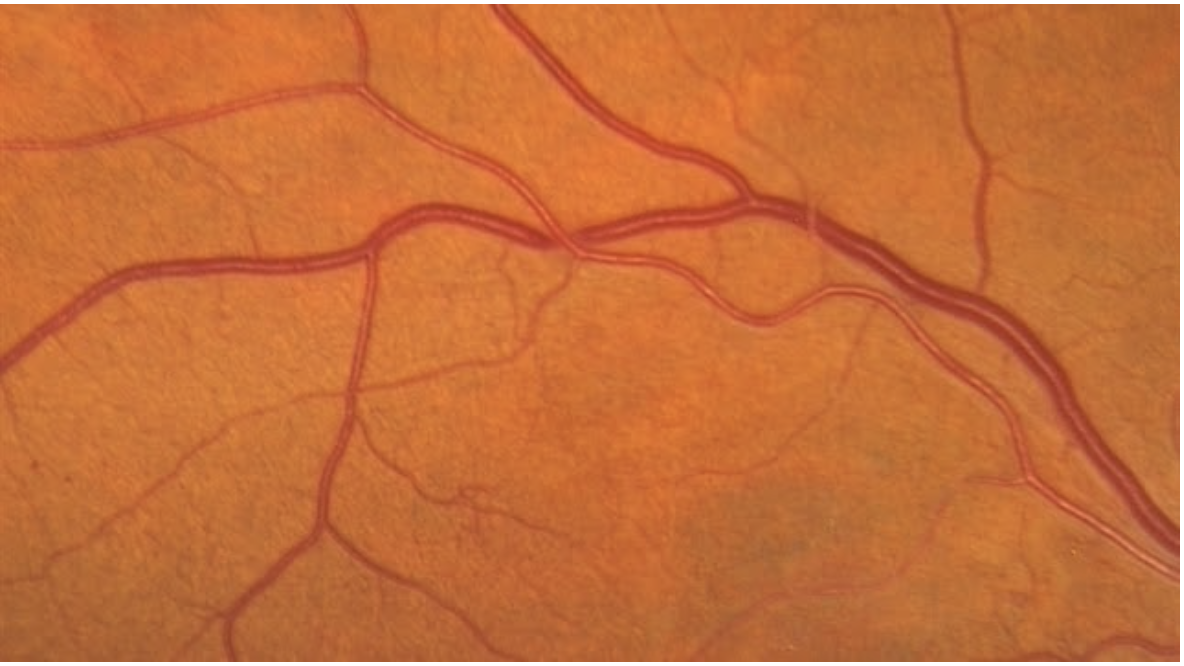


Image is author owned

|  | Check his blood sugar |
| --- | --- |
|  | Check his blood pressure |
|  | Check his intracranial pressure |
|  | Reassure him that he has a normal eye exam |
