# Supplementary material for: A Novel Approach to Teaching Fundoscopy Using a Virtual Format
Source: MedEdPORTAL. 2022 May 27;18:11252. doi: 10.15766/mep_2374-8265.11252 (PMC9135915; doi:10.15766/mep_2374-8265.11252)

How to Look at a Retina – a systematic approach to the fundus exam

REMEMBER: the best way to do any physical exam is with an organized and systematic approach. By using the same method for examination and evaluation, it is more likely that you will find all the pathologies present, instead of just the ones that are most obvious.

5 main parts of the fundus exam: the view, the nerve, the vessels, the macula and the periphery

- The view: is everything that we are looking THROUGH clear and giving us a sharp image?
    - The lens, the cornea, the anterior chamber, the lens, the vitreous

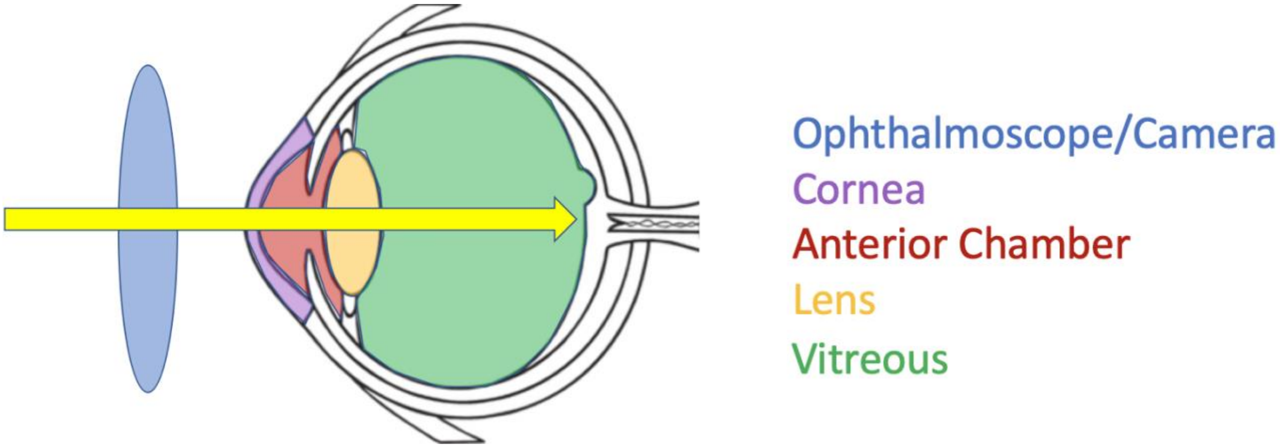

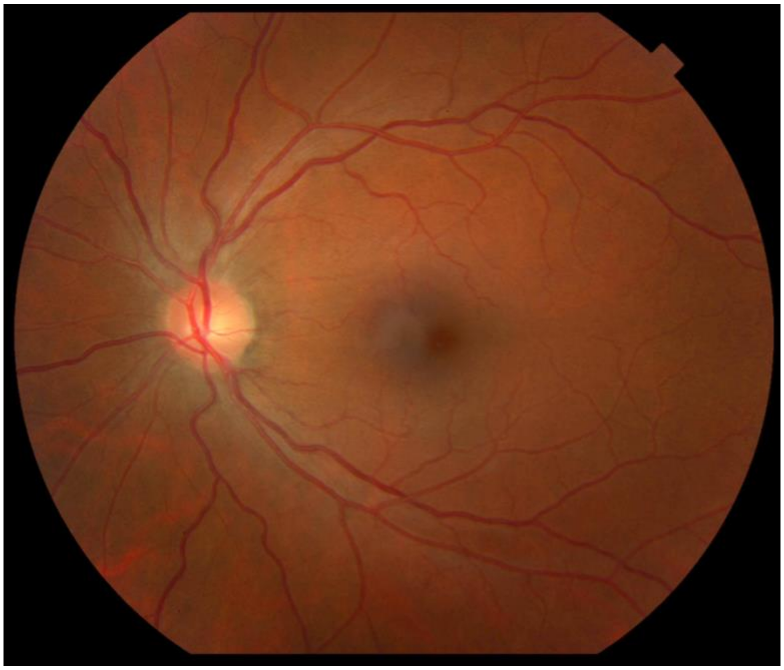

- The nerve: look at the size (1.5mm), shape (round), color (light pink/orange), margins (sharp and visible all around), cupping (0.2-0.5 is normal)

- The vessels: They should be no attenuation, dilation plaques, bleeds, cotton wool spots, neovascularization

- The macula: Should be flat, without pigmentary mottling, pallor or atrophy. There should be no bleeding or deposits.

- The periphery: should be relatively uniform without areas of atrophy, degeneration, deposits, bleeds, tears or pigmentary mottling/changes

SELF PRACTICE TIME

Take a swing at being an ophthalmologist. Often, ophthalmology providers will record their findings in the form of pictures that supplement their written notes. Try drawing your findings and describing them in the spaces provided. Check the answers in the back. For example:

Media/View: _*clear, all aspects of the view are sharp*

Nerve:

Shape: __*Round*____________

Margins: __*Sharp*___________

Color:_____*Pale orange*____________

C:D ratio: ____*0.3*__________

Vessels: __*Veins appear somewhat engorged with bleeds in the mid periphery*__________

Macula: ____*Some pigmentary mottling, shiny yellow refractile deposits temporally*_____

Periphery: ___*Somewhat evenly spaced yellow atrophic vs scarred looking circles*_________

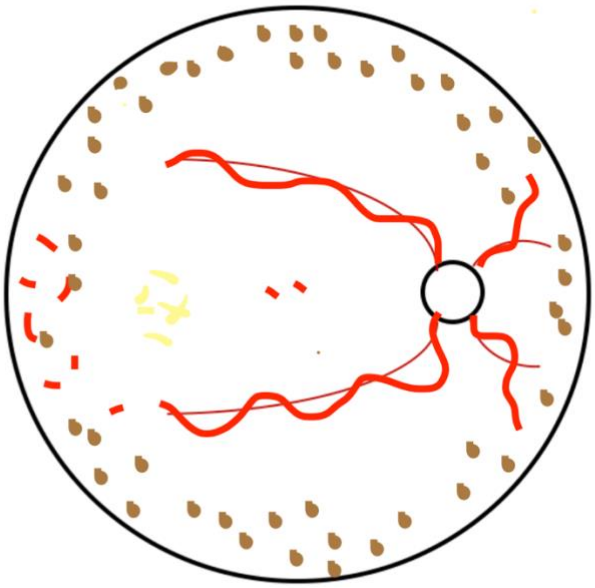

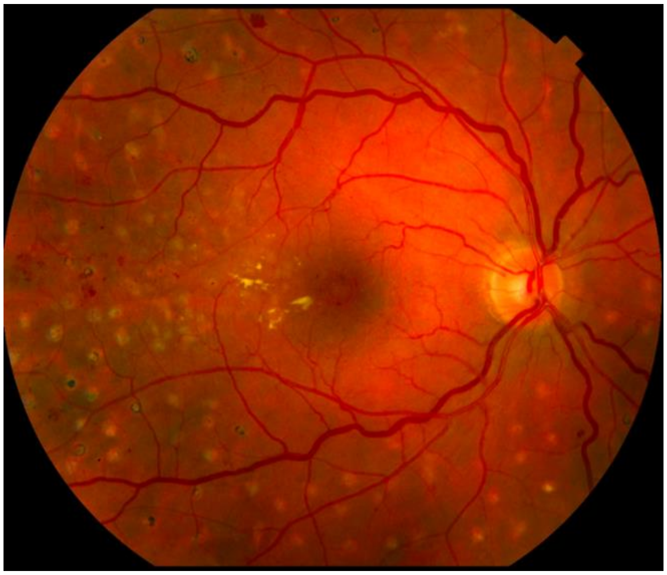

Media/View: ____

Nerve:

Shape: ____

Margins: ____

Color: ____

C:D ratio: ____

Vessels:____

Macula: ____

Periphery: ____

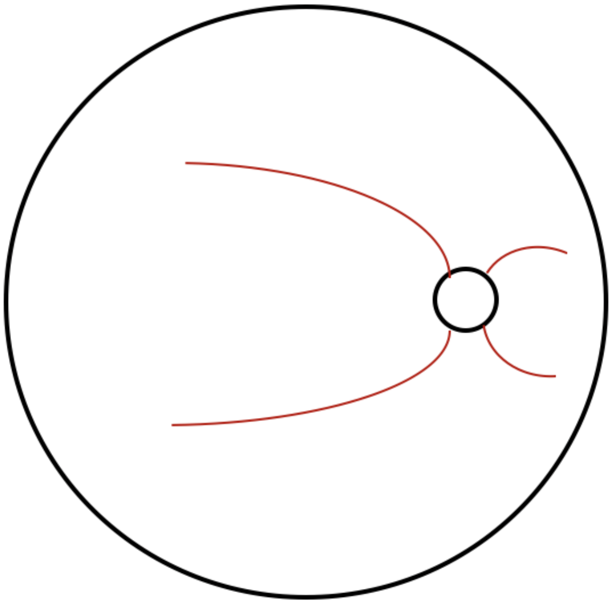

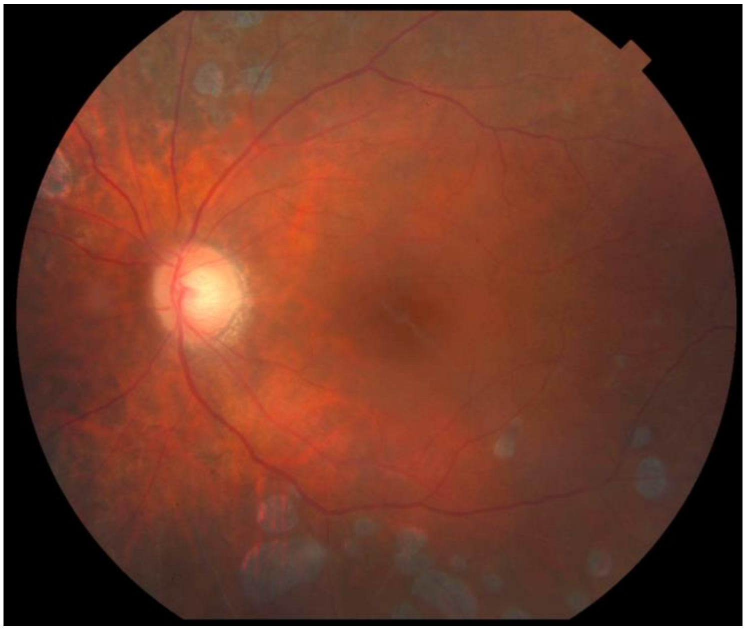

Media/View: ____
Nerve:

Shape: ____

Margins: ____

Color: ____

C:D ratio: ____

Vessels:____

Macula: ____

Periphery: ____

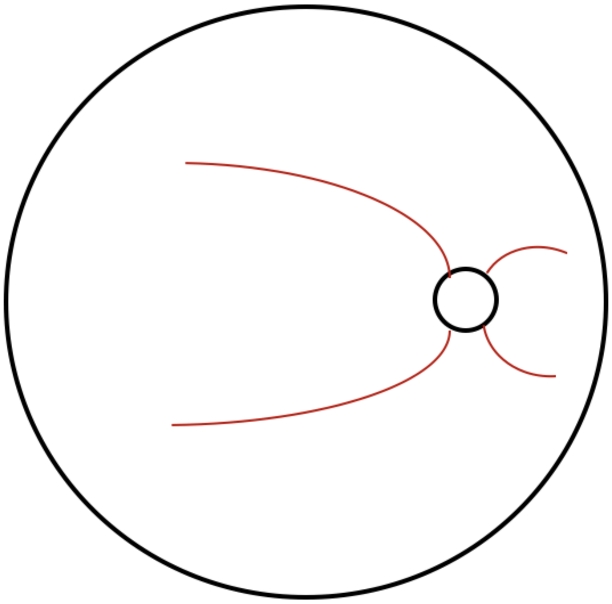

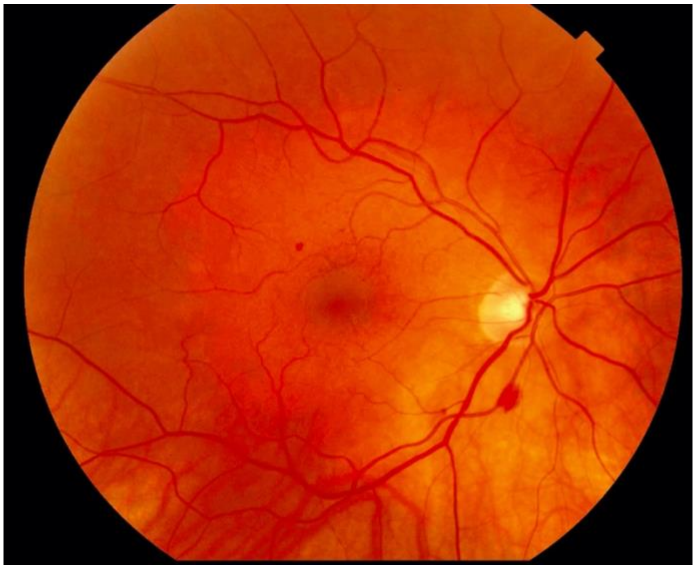

Media/View: ____

Nerve:

Shape: ____

Margins: ____

Color: ____

C:D ratio: ____

Vessels:____

Macula: ____

Periphery: ____

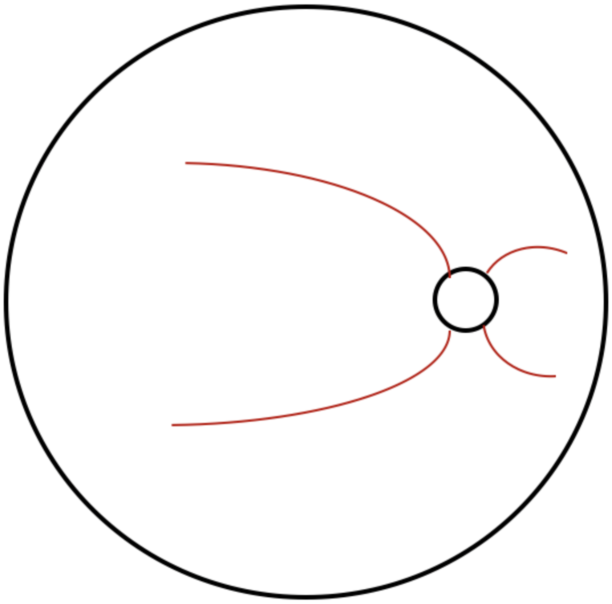

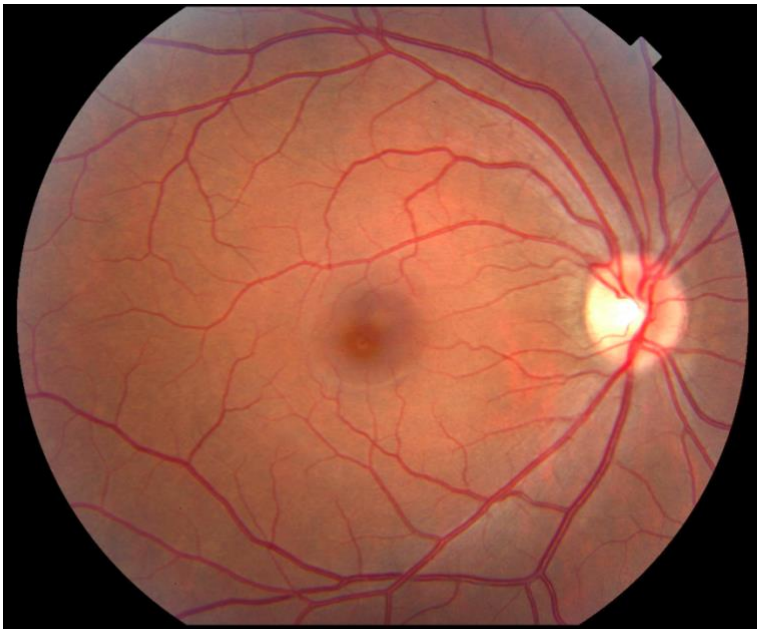

Media/View: ____

Nerve:

Shape: ____

Margins: ____

Color: ____

C:D ratio: ____

Vessels: ____

Macula: ____

Periphery: ____

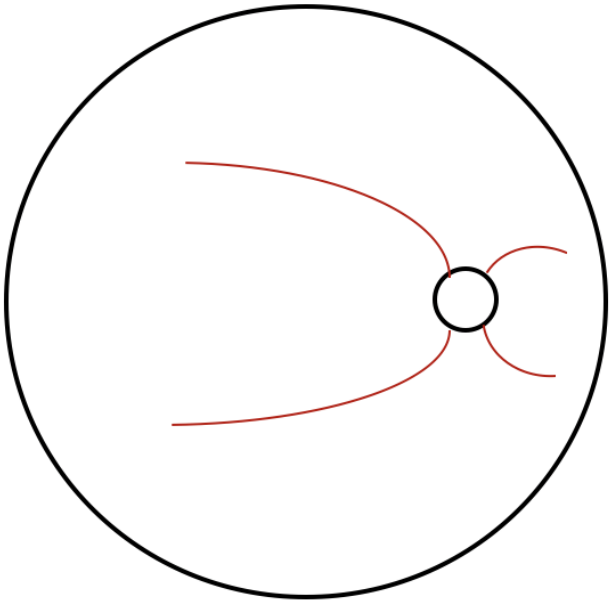

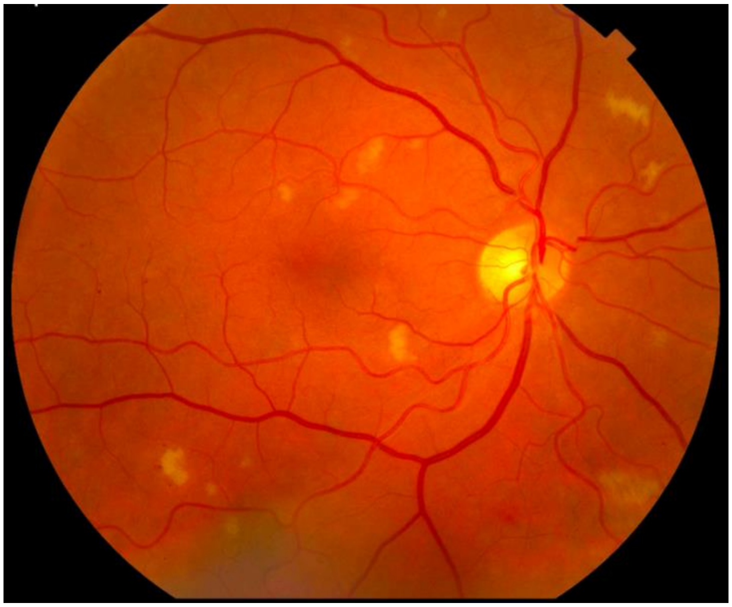

Media/View: ____

Nerve:

Shape: ____

Margins: ____

Color: ____

C:D ratio: ____

Vessels:____

Macula: ____

Periphery: ____

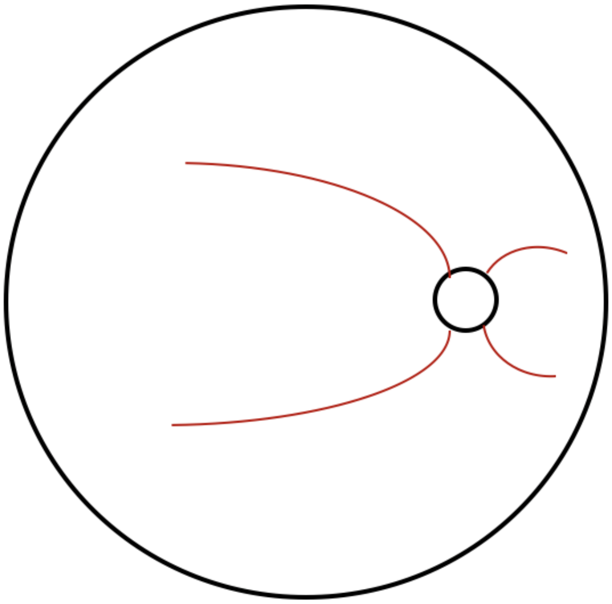

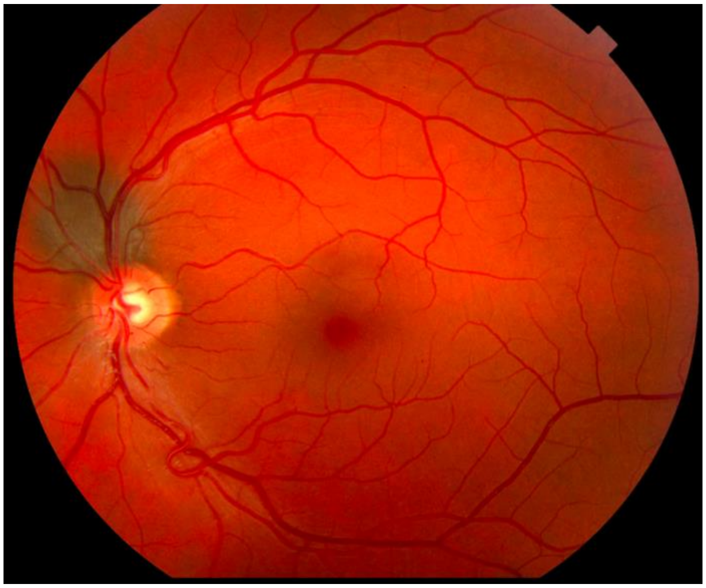

Media/View: ____

Nerve:

Shape: ____

Margins: ____

Color: ____

C:D ratio: ____

Vessels:____

Macula: ____

Periphery: ____

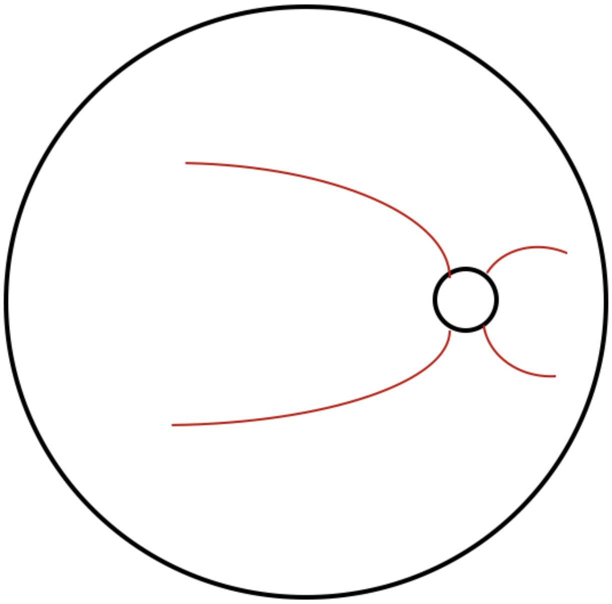

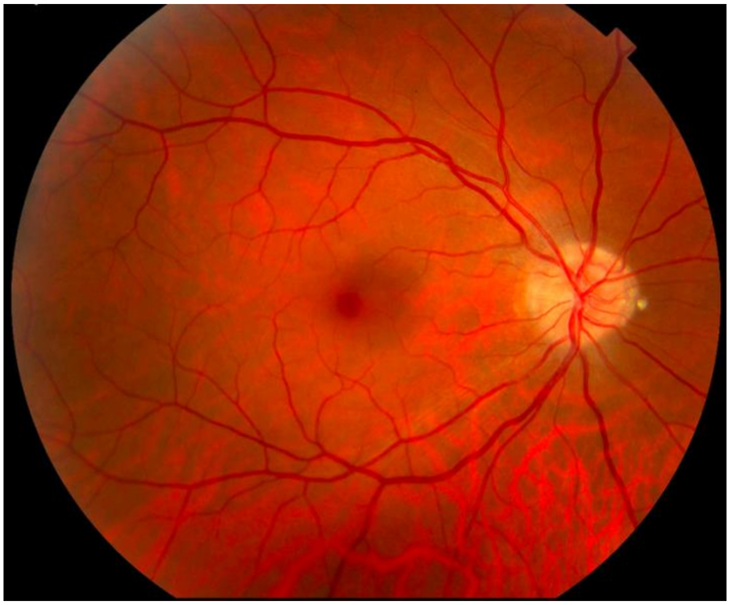

Media/View: ____

Nerve:

Shape: ____

Margins: ____

Color: ____

C:D ratio: ____

Vessels:____

Macula: ____

Periphery: ____

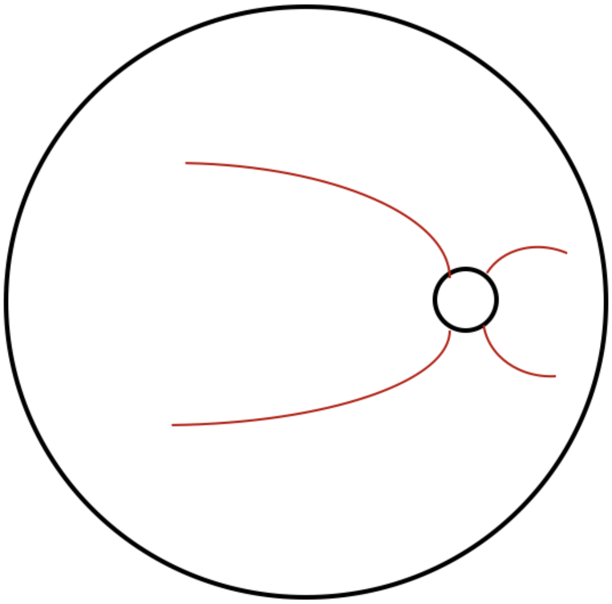

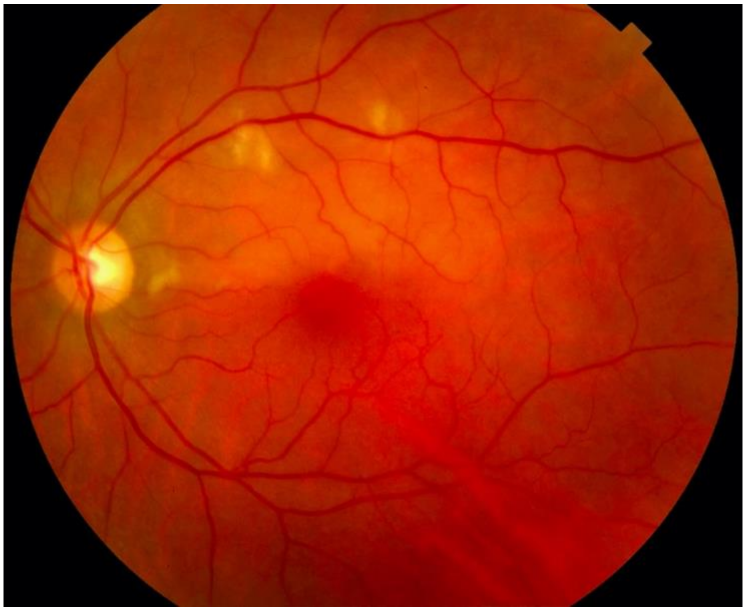

Media/View: ____

Nerve:

Shape: ____

Margins: ____

Color: ____

C:D ratio: ____

Vessels:____

Macula: ____

Periphery: ____

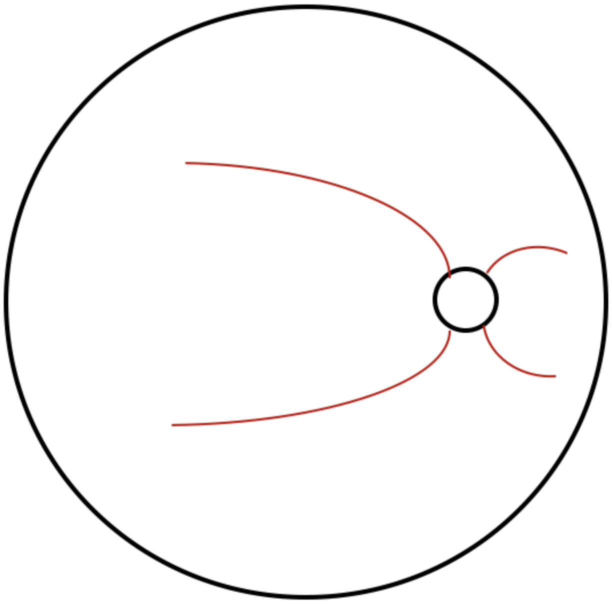

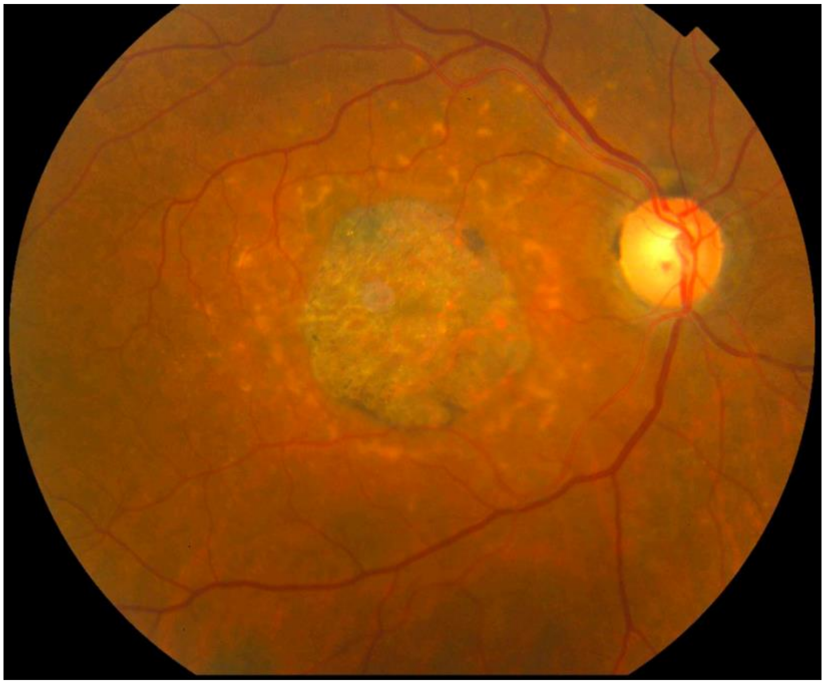

Page 3:
Media/View: mildly hazy view, though all structures visible
Nerve: Shape – round; Margins – sharp, but with ring of yellow discoloration surrounding, especially temporally; Color – pale pink C:D ratio – 0.4
Vessels: Somewhat attenuated/thin
Macula: no mottling or pigment changes, no yellow deposits or bleeds
Periphery: diffuse grey appearing circular holes

Page 4:
Media/View: clear
Nerve: Shape – round; Margins – sharp; Color – pale orange C:D ratio – 0.3
Vessels: flame shaped hemorrhage along the inferior arterial arcade, somewhat dilated venules, somewhat attenuated looking arteries
Macula: diffuse dots of heme within the macula
Periphery: no deposits or pigmentary changes

Page 5:
Media/View: clear
Nerve: Shape – round; Margins – sharp; Color –possible temporal pallor, otherwise pale orange C:D ratio – 0.5 with possible notching at supero-temporal edge of cup
Vessels: no bleeds, attenuation or AV nicking
Macula: no pigment mottling, good foveal reflex, no deposits or bleeds
Periphery: no deposits or pigmentary changes

Page 6:
Media/View: clear
Nerve: Shape – round; Margins – sharp; Color –pale orange C:D ratio – 0.2
Vessels: fluffy appearing yellow lesions (cotton wool spots) along superior and inferior arcades, copper wiring of arterioles, few bleeds inferiorly, AV nicking throughout
Macula: no pigment mottling, no deposits or bleeds
Periphery: Possible pigmented lesion inferiorly

Page 7:
Media/View: clear
Nerve: Shape – round; Margins – sharp; Color –pale orange C:D ratio – 0.4
Vessels: small area of arteriolar thinning along inferior arcade
Macula: no pigment mottling, no deposits or bleeds
Periphery: Pigmented lesion near the optic disc supero-nasally

Page 8:
Media/View: clear
Nerve: Shape – round, with lumpy appearance; Margins – somewhat blurred by apparent ring of yellowish disoloration; Color –pale orange; C:D ratio – 0.1
Vessels: no bleeds, attenuation or AV nicking
Macula: no pigment mottling, good foveal reflex, no deposits or bleeds
Periphery: no deposits or pigmentary changes

Page 9:
Media/View: clear
Nerve: Shape – round; Margins – sharp; Color –possible temporal pallor, otherwise pale orange C:D ratio – 0.5 with possible notching at supero-temporal edge of cup
Vessels: fluffy appearing yellow lesions (cotton wool spots) along superior arcade
Macula: mild palor superiorly, no pigment mottling, good foveal reflex, no deposits or bleeds
Periphery: retinal pallor along superior arcade

Page 10:
Media/View: clear
Nerve: Shape – round; Margins – sharp, but with ring of grey/yellow discoloration surrounding; Color –pale orange C:D ratio – 0.5; possible small bleed within the nerve
Vessels: Attenuated arteries, somewhat dilated veins, AV nicking
Macula: Large geographic pigmentary change with grey/atrophic area; oft white appearing deposits in macula and surrounding retina
Periphery: no deposits or pigmentary changes

Media/View: ____

Nerve:

Shape: ____

Margins: ____

Color: ____

C:D ratio: ____

Vessels:____

Macula: ____

Periphery: ____

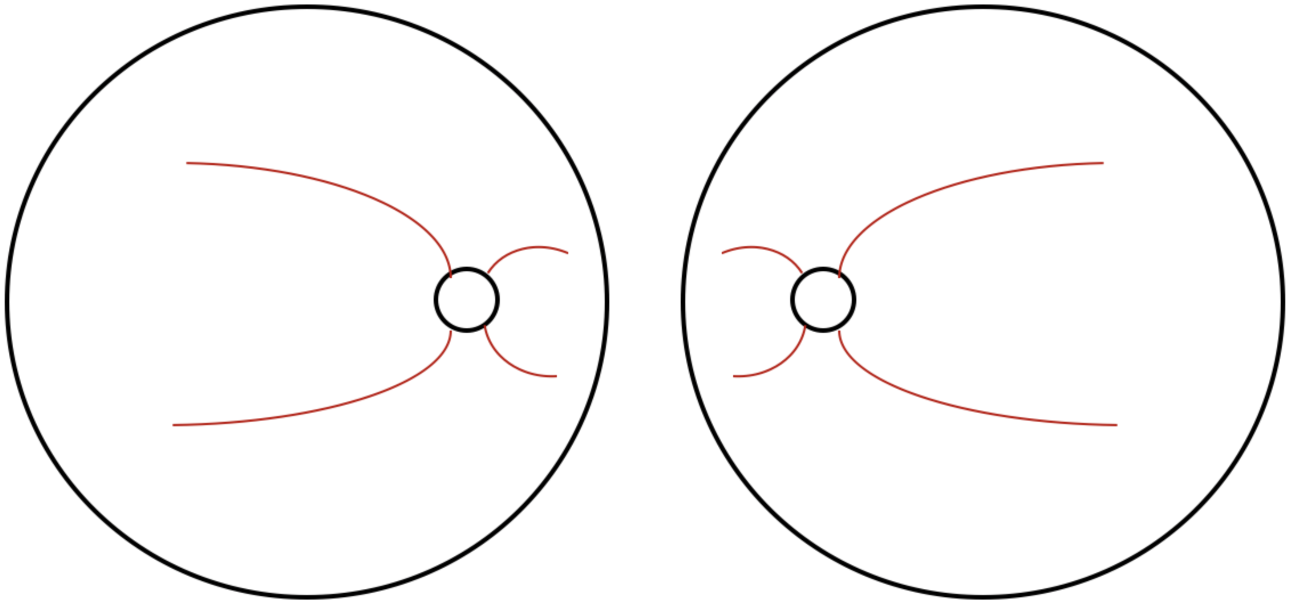

Media/View: ____

Nerve:

Shape: ____

Margins: ____

Color: ____

C:D ratio: ____

Vessels:____

Macula: ____

Periphery: ____

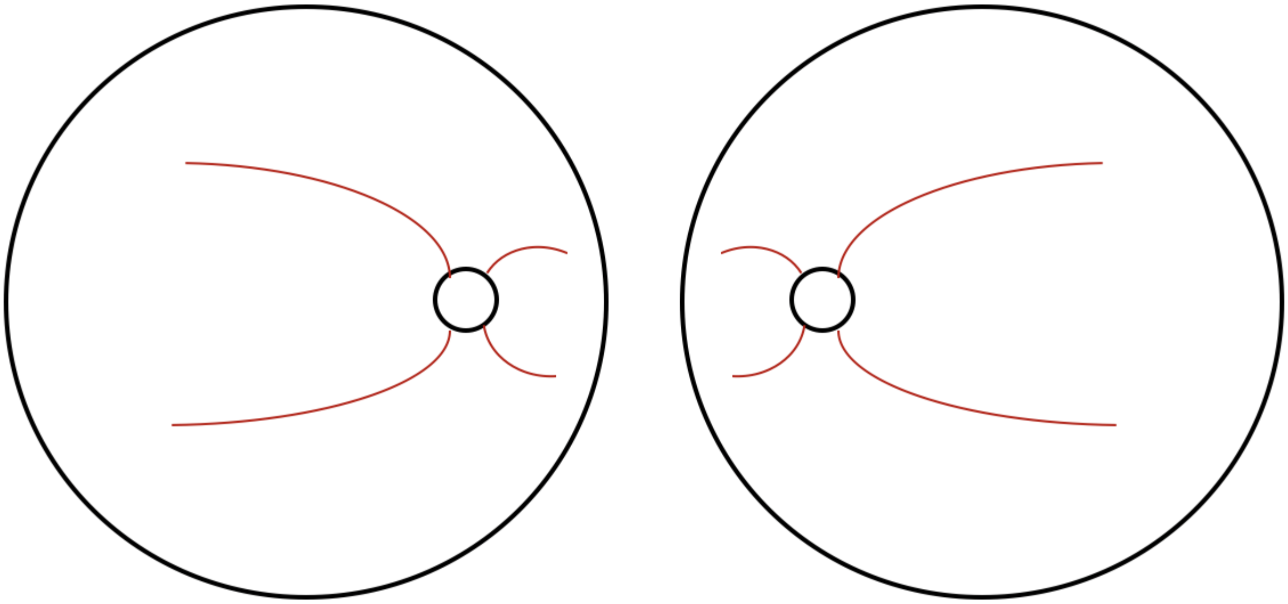

Media/View: ____

Nerve:

Shape: ____

Margins: ____

Color: ____

C:D ratio: ____

Vessels: ____

Macula: ____

Periphery: ____

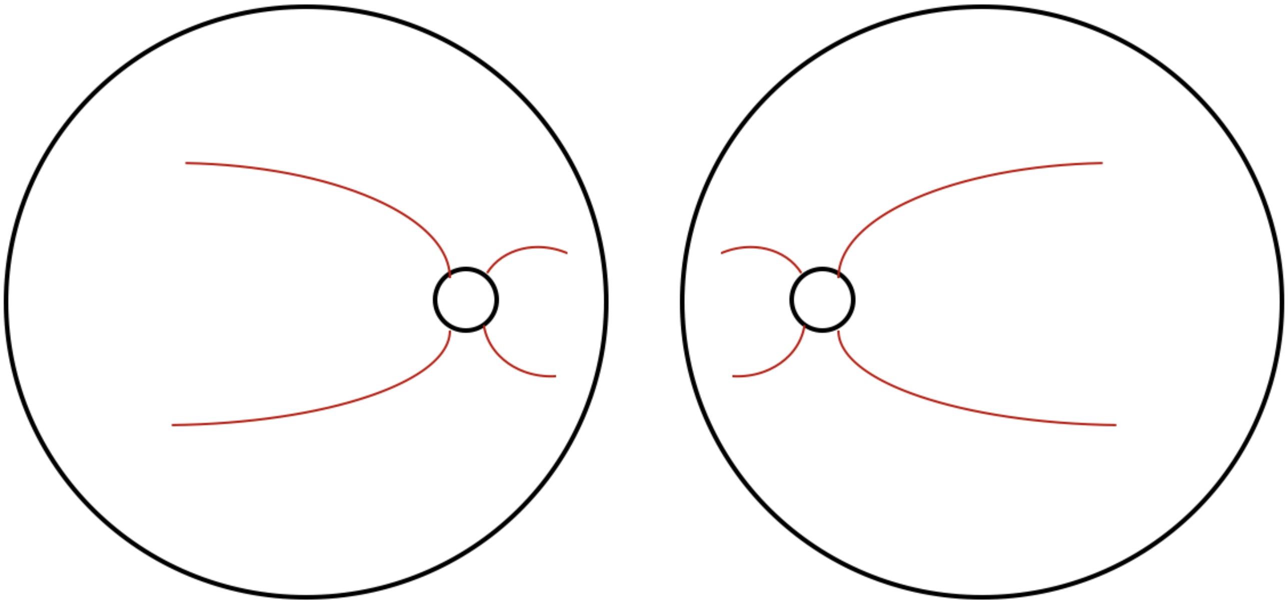

Supplement: Supplementary file 1 — Pretest.docxSlide Deck.pptxPosttest.docxPostworkshop Handout.pdfMedical Student Session Leader Survey.docx [file mep_2374-8265.11252-s001.zip › D. Postworkshop Handout.pdf]
