# Supplementary material for: A Novel Approach to Teaching Fundoscopy Using a Virtual Format
Source: MedEdPORTAL. 2022 May 27;18:11252. doi: 10.15766/mep_2374-8265.11252 (PMC9135915; doi:10.15766/mep_2374-8265.11252)
Supplement: Supplementary file 1 — Pretest.docxSlide Deck.pptxPosttest.docxPostworkshop Handout.pdfMedical Student Session Leader Survey.docx [file mep_2374-8265.11252-s001.zip › E. Medical Student Session Leader Survey.docx]

Medical Student Session Leader Survey

Thank you for your participation in the virtual ophthalmoscopy teaching sessions. Please complete this survey regarding your experience as an educator during this activity.

What is your current year in medical school?

|  | 1^st^ year |
| --- | --- |
|  | 2^nd^ year |
|  | 3^rd^ year |
|  | 4^th^ year |
|  | Research/Gap year |

How many sessions did you lead?

|  | One |
| --- | --- |
|  | Two |
|  | Three |

Please describe any prior teaching experiences:

____________________________________________________________________________

Please describe your prior experience in ophthalmology.

____________________________________________________________________________

I now have a greater interest in teaching/medical education.

|  | 1 | 2 | 3 | 4 | 5 | 6 | 7 |  |
| --- | --- | --- | --- | --- | --- | --- | --- | --- |
| Strongly disagree |  |  |  |  |  |  |  | Strongly agree |

I now have a greater interest in ophthalmology.

|  | 1 | 2 | 3 | 4 | 5 | 6 | 7 |  |
| --- | --- | --- | --- | --- | --- | --- | --- | --- |
| Strongly disagree |  |  |  |  |  |  |  | Strongly agree |

It is important for medical students to have some education in the interpretation of fundus photos.

|  | 1 | 2 | 3 | 4 | 5 | 6 | 7 |  |
| --- | --- | --- | --- | --- | --- | --- | --- | --- |
| Strongly disagree |  |  |  |  |  |  |  | Strongly agree |

Interpretation of fundus photos is more relevant than learning how to perform direct ophthalmoscopy for the non-ophthalmologist.

|  | 1 | 2 | 3 | 4 | 5 | 6 | 7 |  |
| --- | --- | --- | --- | --- | --- | --- | --- | --- |
| Strongly disagree |  |  |  |  |  |  |  | Strongly agree |

I now feel more comfortable teaching fellow medical students.

|  | 1 | 2 | 3 | 4 | 5 | 6 | 7 |  |
| --- | --- | --- | --- | --- | --- | --- | --- | --- |
| Strongly disagree |  |  |  |  |  |  |  | Strongly agree |

My confidence in teaching and answering students’ questions increased over the three sessions (if applicable).

|  | 1 | 2 | 3 | 4 | 5 | 6 | 7 |  |
| --- | --- | --- | --- | --- | --- | --- | --- | --- |
| Strongly disagree |  |  |  |  |  |  |  | Strongly agree |

I now feel more comfortable interpreting a fundus photo.

|  | 1 | 2 | 3 | 4 | 5 | 6 | 7 |  |
| --- | --- | --- | --- | --- | --- | --- | --- | --- |
| Strongly disagree |  |  |  |  |  |  |  | Strongly agree |

I now feel more comfortable with the systematic approach to interpretation of a medical study.

|  | 1 | 2 | 3 | 4 | 5 | 6 | 7 |  |
| --- | --- | --- | --- | --- | --- | --- | --- | --- |
| Strongly disagree |  |  |  |  |  |  |  | Strongly agree |

I now have a better understanding of glaucoma.

|  | 1 | 2 | 3 | 4 | 5 | 6 | 7 |  |
| --- | --- | --- | --- | --- | --- | --- | --- | --- |
| Strongly disagree |  |  |  |  |  |  |  | Strongly agree |
|  | 1 | 2 | 3 | 4 | 5 | 6 | 7 |  |
| Strongly disagree |  |  |  |  |  |  |  | Strongly agree |

I now have a better understanding of what a cup to disk ratio represents.

|  | 1 | 2 | 3 | 4 | 5 | 6 | 7 |  |
| --- | --- | --- | --- | --- | --- | --- | --- | --- |
| Strongly disagree |  |  |  |  |  |  |  | Strongly agree |

I now have a better understanding of diabetic retinopathy.

|  | 1 | 2 | 3 | 4 | 5 | 6 | 7 |  |
| --- | --- | --- | --- | --- | --- | --- | --- | --- |
| Strongly disagree |  |  |  |  |  |  |  | Strongly agree |

I now have a better understanding of hypertensive retinopathy

|  | 1 | 2 | 3 | 4 | 5 | 6 | 7 |  |
| --- | --- | --- | --- | --- | --- | --- | --- | --- |
| Strongly disagree |  |  |  |  |  |  |  | Strongly agree |

These teaching sessions provide a good introduction to ophthalmology for first year medical students.

|  | 1 | 2 | 3 | 4 | 5 | 6 | 7 |  |
| --- | --- | --- | --- | --- | --- | --- | --- | --- |
| Strongly disagree |  |  |  |  |  |  |  | Strongly agree |

These teaching sessions provide a good introduction to systematic interpretation of medical studies for first year medical students.

|  | 1 | 2 | 3 | 4 | 5 | 6 | 7 |  |
| --- | --- | --- | --- | --- | --- | --- | --- | --- |
| Strongly disagree |  |  |  |  |  |  |  | Strongly agree |

Please share any comments about how your participation as a session leader influenced your understanding of retinal pathologies and your ability to interpret a fundus photo.

____________________________________________________________________________

Please share any comments about how your participation as a session leader influenced your ability to teach fellow medical students.

____________________________________________________________________________

Please share any comments about how your participation as a session leader influenced your ability to collaborate with a large group on a project.

____________________________________________________________________________
